# Developing a toolkit for the assessment and monitoring of musculoskeletal ageing

**DOI:** 10.1093/ageing/afy143

**Published:** 2018-09-07

**Authors:** Graham J Kemp, Fraser Birrell, Peter D Clegg, Daniel J Cuthbertson, Giuseppe De Vito, Jaap H van Dieën, Silvia Del Din, Richard Eastell, Patrick Garnero, Katarzyna Goljanek–Whysall, Matthias Hackl, Richard Hodgson, Malcolm J Jackson, Sue Lord, Claudia Mazzà, Anne McArdle, Eugene V McCloskey, Marco Narici, Mandy J Peffers, Stefano Schiaffino, John C Mathers

**Affiliations:** 1 Department of Musculoskeletal Biology, Faculty of Health and Life Sciences, Institute of Ageing and Chronic Disease (IACD), University of Liverpool, William Duncan Building, 6 West Derby Street, Liverpool, UK; 2 Institute of Cellular Medicine, Musculoskeletal Research Group, Newcastle University, Newcastle upon Tyne, UK; 3 Department of Human Movement Sciences, VU University Amsterdam, Amsterdam Movement Sciences, Van der Boechorststraat 9, Amsterdam, The Netherlands; 4 Clinical Ageing Research Unit, Institute of Neuroscience/Newcastle University Institute for Ageing, Campus for Ageing and Vitality, Newcastle University, Newcastle upon Tyne, UK; 5 Mellanby Centre for Bone Research, University of Sheffield, Sheffield, UK; 6 Division of Bone Diseases, Geneva University Hospital and Faculty of Medicine, 1205 Geneva, Switzerland; 7 TAmiRNA GmbH, Muthgasse 18, Vienna, Austria; 8 Centre for Imaging Sciences, University of Manchester, Stopford Building, Oxford Road, Manchester, UK; 9 Department of Mechanical Engineering & INSIGNEO Institute for in silico Medicine, University of Sheffield, Sheffield, UK; 10 MRC-ARUK Centre of Excellence for Musculoskeletal Ageing Research, Derby Royal Hospital, Uttoxeter Road, Derby, UK; 11 Venetian Institute of Molecular Medicine (VIMM), Via Orus 2, Padova, Italy; 12 School of Public Health, Physiotherapy and Sports Science, Institute for Sport and Health, University College Dublin, Belfield, Dublin, Ireland; 13 Human Nutrition Research Centre, Institute of Cellular Medicine and Newcastle University Institute for Ageing, Newcastle University, Newcastle upon Tyne, UK; 14 The MRC-Arthritis Research UK Centre for Integrated Research into Musculoskeletal Ageing (CIMA)

**Keywords:** biomarkers, bone, cartilage, healthy ageing, musculoskeletal ageing, skeletal muscle

## Abstract

The complexities and heterogeneity of the ageing process have slowed the development of consensus on appropriate biomarkers of healthy ageing. The Medical Research Council–Arthritis Research UK Centre for Integrated research into Musculoskeletal Ageing (CIMA) is a collaboration between researchers and clinicians at the Universities of Liverpool, Sheffield and Newcastle. One of CIMA’s objectives is to ‘Identify and share optimal techniques and approaches to monitor age-related changes in all musculoskeletal tissues, and to provide an integrated assessment of musculoskeletal function’—in other words to develop a toolkit for assessing musculoskeletal ageing. This toolkit is envisaged as an instrument that can be used to characterise and quantify musculoskeletal function during ‘normal’ ageing, lend itself to use in large-scale, internationally important cohorts, and provide a set of biomarker outcome measures for epidemiological and intervention studies designed to enhance healthy musculoskeletal ageing. Such potential biomarkers include: biochemical measurements in biofluids or tissue samples, *in vivo* measurements of body composition, imaging of structural and physical properties, and functional tests. This review assesses candidate biomarkers of musculoskeletal ageing under these four headings, details their biological bases, strengths and limitations, and makes practical recommendations for their use. In addition, we identify gaps in the evidence base and priorities for further research on biomarkers of musculoskeletal ageing.

## Introduction

### Biomarkers of ageing

Ageing is associated with the accumulation of damage to all the macromolecules within and outside cells leading to progressively more cellular and tissue defects and resulting in age-related frailty, disability and disease [1]. There is substantial inter-individual variability in the ageing process, so that biological age can differ considerably from chronological age [2]. However, the complexities and heterogeneities of the ageing process have made it difficult to define and to measure ageing, and this has slowed the development of consensus on appropriate biomarkers.

Despite the limited consensus on biomarkers of ageing, Butler and colleagues have usefully defined three criteria for such markers [3]: the biomarker should predict the outcome of a wide range of age-sensitive tests in multiple physiological and behavioural domains, in an age-coherent way, and do so better than chronological age; it should predict remaining longevity at an age at which 90% of the population is still alive, and do so for most of the specific illnesses that afflict the species under study; and its measurement should not alter life expectancy or the outcome of subsequent age-sensitive tests.

Building on the concept of the healthy ageing phenotype (HAP) introduced by Franco *et al.* [4], Lara *et al*. proposed a pragmatic set of measures of the HAP designed for use in community-based intervention studies aiming to promote healthy ageing [5]. In a parallel exercise, a working group from five UK universities, funded by the Medical Research Council (MRC), developed a panel of biomarkers of healthy ageing which may have utility in epidemiological studies of human ageing, in health surveys of older people, and as outcomes in intervention studies that aim to promote healthy ageing [6]. Based on comprehensive reviews of the literature, the working group identified a series of candidate markers across five domains in which function declines with ageing, viz. cognition, physical capability, physiological function, and endocrine and immune function [6]. The predictive value of various putative markers of ageing remains uncertain. However, trajectories of healthy ageing, obtained from estimates of the HAP measured three times over nearly 10 years in participants in the English Longitudinal Study of Ageing, showed the expected secular decline and demonstrated that these trajectories were socioeconomically patterned [7]. In addition, lifestyle factors known to influence ageing, including smoking and physical activity, were associated with trajectories of healthy ageing (Tampubolon, 2016). The importance of using panels of biomarkers when assessing ageing was illustrated using blood-based biomarkers in participants in the Long Life Family Study, where age-related changes in individual biomarkers did not necessarily correlate with disease risk or functional decline [8].

Although the need has been identified [9], to date there appears to have been no attempt to develop a specific set of biomarkers of ageing of the musculoskeletal system. However, in the USA the recent development of the NIH toolbox for assessment of neurological and behavioural function [10] provides several relevant measures. These include five practical, low-cost tests of motor function across five sub-domains: dexterity—9-hole pegboard; strength—hand grip strength; balance—standing balance; locomotion—4-metre walking test, and endurance—2-min walking test. In the Netherlands, Englefreit *et al*. proposed a panel of biochemical markers of ageing for use in longitudinal studies, with the criterion that: ‘… ideally, the sought biomarkers should be specific for changes that occur in a particular organ in virtually all humans as they age’ [11]. In respect of musculoskeletal ageing, they identified eight potential biomarkers: three for collagen—N- and C-telopeptide cross-links of type I collagen, procollagen type I N-terminal propeptide and C-terminal propeptide; three for bone–bone-specific alkaline phosphatase, osteoclast-derived tartrate-resistant acid phosphatase 5b, and osteocalcin and one each for cartilage and muscle—cartilage oligomeric matrix protein (COMP) and irisin, respectively [11].

The heterogeneity in types of biomarkers proposed by each of these groups reflects the different objectives of the proposers and the complexity of the ageing process, and emphasises that no single biomarker of ageing is likely to be appropriate for all tissues or in all circumstances.

### Towards a toolkit for assessing musculoskeletal ageing

The MRC—Arthritis Research UK Centre for Integrated research into Musculoskeletal Ageing (CIMA)^1^ is a collaboration between researchers and clinicians at the Universities of Liverpool, Sheffield and Newcastle. One of CIMA’s objectives is to ‘identify and share optimal techniques and approaches to monitor age-related changes in all musculoskeletal tissues, and to provide an integrated assessment of musculoskeletal function’—in other words, to develop a toolkit for assessing musculoskeletal ageing. The current report outlines the result of that activity and extends the outcomes of a dedicated workshop, held in Manchester, the UK on 25 May 2016, with a panel of experts from the UK and European institutions with well-established track records of research into musculoskeletal ageing. The ambitions for the toolkit are that it should: characterise and quantify musculoskeletal function over multiple decades, i.e. during ‘normal’ ageing; facilitate epidemiological assessment of musculoskeletal decline, provide a set of outcome measures for intervention studies (using, e.g. drugs or lifestyle) designed to enhance healthy musculoskeletal ageing and become the protocol of choice, adopted by multiple large-scale, internationally important cohorts.

Workshop participants were tasked with defining a framework for the selection of biomarkers of ageing that are relevant to the multiple tissues of the musculoskeletal system, are distinct from markers of disease, change with age and are sensitive to intervention.

Recognising that research on biomarkers relevant to specific musculoskeletal tissues and biomarkers of different types is not all at the same stage of development and validation, workshop participants were asked to distinguish between immediately useful biomarkers and those of the next generation when making recommendations for the proposed toolkit. Following the workshop, the draft recommendations and supporting text were circulated to the participants and to CIMA colleagues for comment and additions. This report is the result of that process.

### Aims and structure of this report

This report considers candidate biomarkers of musculoskeletal ageing under four headings:

- Biochemical biomarkers
- Body - composition biomarkers
- Imaging assessments
- Functional assessments, e.g. of muscle strength.

In each case, we begin with a summary of recommendations for the toolkit and go on to provide further detail on the recommended approaches, their biological basis, and strengths and limitations. In the final discussion and conclusions section, we summarise the overall recommendations and identify key priorities for research on biomarkers of musculoskeletal ageing.

## Biochemical biomarkers of musculoskeletal ageing

Biochemical biomarkers are markers measurable *in situ* or *ex vivo* in biofluid samples and tissue biopsies (e.g. precursor, product, enzyme, metabolite or regulatory molecule such as non-coding RNA) that are produced in, or released from, a tissue and that are reasonably specific for a characteristic process or cell in that tissue.

### Bone biochemical biomarkers

#### Bone biochemical biomarkers—recommendations for the toolkit

Two well-established serum markers of bone turnover are recommended for the toolkit:

- N-terminal propeptide of type I procollagen (PINP), which has little diurnal rhythm and shows a large increase during fracture healing.
- C-terminal cross-linked telopeptide of collagen type I (CTX, also known as CTX-I), which shows a marked diurnal rhythm with highest concentrations in the early morning and a nadir in the afternoon.

Five further markers of bone turnover are potential future candidates: osteocalcin; bone alkaline phosphatase; N-terminal cross-linked telopeptide of collagen type I (NTX); carboxy-terminal cross-linked telopeptide of type I collagen generated by matrix metalloproteinases (ICTP) and tartrate-resistant acid phosphatase isoform 5b (TRACP5b, also known as TRACP5b).

#### Bone turnover markers

There are no biochemical markers of bone mass or density *per se*. Bone turnover markers comprise markers of bone formation: the osteoblast matrix protein, osteocalcin; the C- and N-terminal pro-peptides of type I procollagen (PICP, PINP), and the enzyme, bone alkaline phosphatase; and markers of bone resorption: the collagen degradation products, pyridinium cross-links of collagen, deoxypyridinoline (DPD), CTX-I, NTX-I, and ICTP, and the enzyme TRACP5b.

In older women, high levels of these markers are associated with greater fracture risk, faster rates of bone loss, and a greater response to anti-resorptive and anabolic treatment, while in both men and women they decrease with anti-resorptive therapy and increase with anabolic therapy. They may also be used in assessing growth, adaptation of the maternal skeleton to pregnancy, and the effects of drugs and of metabolic bone diseases including osteoporosis, Paget’s disease and osteomalacia.

The TRIO study, a 2-year open-label parallel randomised control trial of oral ibandronate, alendronate and risedronate in 172 post-menopausal women, designed to compare the drug effects on bone turnover and bone mineral density, highlighted CTX and PINP as the most responsive markers of bone resorption and formation, respectively [12]. Least significant change estimates identified similar results across three further studies [13–15], while signal-to-noise ratio determination in the TRIO study confirmed CTX and PINP as optimal reference markers. A position paper of the International Osteoporosis Foundation and International Federation of Clinical Chemistry and Laboratory Medicine has also endorsed serum CTX and PINP as reference markers [16].

It is worth noting that individual bone turnover markers may behave differently in particular circumstances. For example, inhibition of the bone remodelling protease, cathepsin K, reduced urinary CTX throughout 24 months of treatment, while the levels of other bone resorption markers (ICTP, TRACP5b) remained similar to those with placebo or were increased [17].

The different behaviours of these markers reflect their production within the cycle of bone formation and resorption. Of the markers of bone resorption, ICTP is generated by matrix metalloproteinase activity, TRACP5b reflects osteoclast number, and CTX results from cathepsin K activity. Of the markers of bone formation, PINP is cleared by liver and reflects synthesis of type I collagen, osteocalcin is cleared by kidney and reflects late osteoblast activity, and bone alkaline phosphatase is cleared by liver and reflects early osteoblast activity.

All these bone turnover markers have been externally validated by reference to the gold standard approaches of bone histomorphometry and radiotracer kinetics [18, 19].

#### Other potential bone biochemical markers—local and systemic regulators

In the future, consideration will be given to adding to the toolkit assays for regulators of bone turnover, especially those that can be measured in the circulation. These include sclerostin and Dickkopf-1, which act by blocking the canonical Wnt signalling pathway [20, 21], and periostin, a protein secreted by periosteal osteoblasts and osteocytes that may also work through the Wnt signalling pathway and play a role in periosteal apposition [22]. Regulators of bone resorption, e.g. RANKL: receptor activator of nuclear factor κB ligand [23], and osteoprotegerin, could be of use. However, results with such local regulators have so far been poor, and much of the basic physiology of the circulating forms is unknown.

### Cartilage biochemical biomarkers

#### Cartilage biochemical markers—no current recommendations for the toolkit

Urinary type II collagen C-telopeptide fragment (CTX-II) and serum COMP are two potential biomarkers of cartilage turnover for which validated commercial assays are available. Currently, these are the best-established markers of increased risk of incidence and progression of knee and hip osteoarthritis (OA) [24, 25]; however, they have not been shown to be reliable markers of collagen ageing *per s*e, independent of OA, and thus cannot be recommended for the toolkit.

#### The search for reliable cartilage biomarkers

The great majority of work on cartilage biomarkers has focused on the detection and monitoring of OA, spurred on by several global initiatives [26, 27], and facilitated by a growing involvement of specialist diagnostic and pharmaceutical companies with active clinical programmes in disease-modifying OA drugs. As with bone, there are no biochemical markers of cartilage properties *per se*, although there are markers of cartilage turnover.

Basic radiography remains the gold standard for assessing joint damage in OA. However, its poor sensitivity and precision precludes early detection of the disease or efficient monitoring of joint damage progression. Thus, the search for biomarkers has focused on two key molecules of the cartilage extracellular matrix: the proteoglycan, aggrecan, and the main matrix protein, type II collagen, together with some selected non-collagenous, non-proteoglycan, proteins, such as COMP. The metabolism of these proteins can be followed non-invasively via the assessment in biological samples of a variety of pro-peptides (for collagen synthesis), degradation products and antigenic epitopes (for collagen and aggrecan degradation) [28, 29]. CTX-II is the best characterised type II collagen fragment used as a marker of cartilage degradation, while several aggrecan-specific neoepitopes have also been studied.

Large epidemiological studies have confirmed that urinary CTX-II and serum COMP are useful for assessing both the progression and incidence of OA in the knee and hip [25, 27, 30–32]. However, there is room for improvement, for example in the standardisation of assays. Moreover, there are doubts about the specificity of these markers. COMP, for example, is also synthesised by synovial and tendon cells, while CTX-II has been shown to increase in parallel with bone biomarkers, and there have been suggestions that it reflects mainly an increase in calcified cartilage tissue [33, 34]. This lack of specificity is highlighted by depression of CTX-II after osteoporosis treatments and an association with disc degeneration [35]. As noted earlier for biomarkers of ageing *per se* [8], ‘multiplexing’ of biochemical marker combinations may be more useful and appropriate than individual biomarkers [33, 36]. For example, Kraus *et al*. have identified several systemic candidate biomarkers that are potential predictors of pain and structural worsening of OA [37].

Distinguishing between the effects of OA and of ageing on cartilage biomarker levels is difficult. There is some evidence of increased collagen turnover with age independent of OA [38, 39]. However, few human studies have investigated changes in collagen markers in the general population without OA, and these have not usually involved careful examination of different joints by X-ray, so it is not known whether apparent increases in biomarkers are an age effect, or simply indicative of a higher prevalence of OA with age.

Age-related collagen molecules such as pentosidine or the d-isomer of aspartic acid are potential markers of aged cartilage matrix, though they are not specific for cartilage tissue when measured in biological fluid [40]. If cartilage specific age-related molecules could be isolated they may be possible future biomarkers of ageing. Recent studies using matrix-assisted laser desorption ionisation mass spectrometry imaging (MALDI-MSI) have identified a tryptic peptide of COMP which was more abundant in non-OA cartilage from old versus young horses. This peptide represents a possible marker of age-related, but not disease-related, changes in cartilage tissue [41]. Whether such peptides could be measured in serum or urine and used as reliable markers of age-related changes in cartilage in humans remains to be determined.

### Muscle biochemical biomarkers

#### Muscle biochemical markers—recommendations for the toolkit

- Serum creatinine may be a reliable biomarker of skeletal muscle mass (with appropriate dietary control).

Potential next-generation muscle biochemical biomarkers include: 3-methylhistidine, type VI collagen, P3NP (the N-terminal peptide of procollagen type III), agrin and growth differentiation factors (GDFs).

#### Established and emerging muscle biomarkers

The loss of muscle mass and function that are associated both with ageing and chronic disease arises from an imbalance between protein synthesis and protein degradation, resulting in muscle atrophy and consequent increases in morbidity and mortality [42]. The term ‘anabolic resistance’ describes the reduced capacity of skeletal muscle of older people to respond to the usual anabolic stimuli, i.e. amino acids and resistance exercise (Cuthbertson *et al.*, 2005; Kumar *et al*., 2009). However, formal measures of the rates of these processes are not markers of changing muscle mass (this case is analogous to the bone turnover markers discussed above in Section 2.1). The search continues for robust biomarkers of muscle ageing that are practicable for diagnosis and therapy monitoring [43–45]. These include the following potential candidate biomarkers.

Serum creatinine concentration and 24-h urinary creatinine excretion appear to be reasonably reliable biomarkers of skeletal muscle mass in humans [46]. Creatinine production rate reflects the total body creatine pool, most of which is intra-myocellular; thus 24-h urinary creatinine output reflects largely muscle mass modulated by variability in muscle creatine concentration, and potentially confounded by variation in dietary intake of creatine in meat and meat products. Serum creatinine concentration is proportional to creatinine production and inversely proportional to creatinine clearance (i.e. essentially, glomerular filtration rate (GFR)); thus, it reflects muscle mass modulated by variability in GFR (which is itself subject to ageing effects [47]). A procedure based on D3-creatine dilution and detection of urinary creatinine enrichment by isotope ratio mass spectrometry could be a useful approach to measure total body skeletal muscle mass [48]; drawbacks are the relatively high cost and limited availability of the necessary equipment, and potential variability in the creatine content of muscle cells.

A recent study has demonstrated the utility of a D_2_O tracer-based approach for quantifying day-to-day changes in muscle protein synthesis which is not feasible with conventional ^13^C tracers that may have relevance for epidemiological or interventional studies [49].

3-Methylhistidine (3MH) concentration in serum and urine is a marker of myofibrillar proteolysis because 3MH is formed through post-translational methylation of specific histidine residues in actin and myosin, and is released when these proteins turn over. However, 3MH is also present in meat and fish [50], so a period of pre-sampling meat restriction is needed to avoid confounding [51].

Type VI collagen is a major constituent of the extracellular matrix of skeletal muscle, and collagen fragments derived from metalloproteinase activity, such as matrix MMP-generated degradation fragment of collagen 6 (C6M) and type VI collagen N-terminal globular domain epitope (IC6), are released into the circulation. Concentrations of a number of these peptides correlate with lean body mass [52] including P3NP—the N-terminal peptide of procollagen type III [53].

Agrin, a protein released from motor neurons at the neuromuscular junction (NMJ), is involved in acetylcholine receptor clustering and NMJ formation and maintenance. Agrin can be cleaved by neurotrypsin, a protease at the NMJ, giving rise to two major C-terminal agrin fragments (CAFs)—CAF110 and CAF22 [54]. These potential biomarkers may lack tissue specificity since increased CAF concentration is seen in patients with chronic heart failure, and increased CAF22 concentration has been associated with kidney failure [55]. However, recent studies have shown that increased serum CAFs concentration can detect skeletal muscle wasting in patients with heart failure [56] and is associated with sarcopenia in older people with multiple morbidities living in the community [57].

Myostatin (GDF8), a negative regulator of muscle growth and mass, is associated with muscle wasting, and has been suggested as a putative marker for muscle atrophy. However, its serum concentration was shown recently to decrease with ageing [58]. In contrast, GDF11 (growth differentiation factor 11), a transforming growth factor-beta superfamily member similar to myostatin and binding the same receptor, does not decline with ageing and is a risk factor for frailty and other morbidities [58]. Serum concentration of GDF15 is increased with ageing and in some pathological conditions, including mitochondrial myopathies, and GDF15 may reflect mitochondrial dysfunctions that accompany the ageing process [59, 60].

### Potential novel biochemical biomarkers: microRNAs, metabolomics and other ‘gestalt’ approaches

This report is focused on a tissue-specific approach to the identification of biomarkers, but there are alternative approaches, including the characterisation of non-coding RNAs (microRNAs (miRNAs) and small nucleolar RNAs (snoRNAs)) [61], use of metabolomics approaches [62] and assay of products of protein oxidation, nitration and glycosylation from urine or serum [63]. Of these, miRNAs are perhaps the most promising, but all need validation in population-based studies.

#### MicroRNAs—no current recommendations for the toolkit

- MiRNAs show promise as biomarkers, but further biological and clinical research is needed to improve our understanding of the potential of these markers before they can be recommended as candidates for the biomarkers of musculoskeletal ageing toolkit.

#### MicroRNAs as biomarkers

MiRNAs are small (typically 22 nucleotides) single-stranded, non-coding RNA species involved in post-transcriptional regulation of gene expression. An individual miRNA may regulate several genes and a given gene may be regulated by multiple miRNAs [64]. Because of their pervasive function in regulation of gene expression, miRNA play a role in ageing and age-related disease, and thus may have significant diagnostic and prognostic potential in researching, monitoring and preventing diseases associated with ageing. Tissue-specific miRNA occur in brain, skeletal muscle and heart, where they act as important regulators of tissue formation and homoeostasis [65]. Since every cell and tissue produces and releases miRNA, they can be detected in both tissue biopsy and blood samples using minimally invasive techniques.

#### MicroRNAs in musculoskeletal turnover

Research into miRNA as potential biomarkers in the musculoskeletal field has investigated links with fracture risk in patients with post-menopausal osteoporosis, diabetic osteopathy and idiopathic osteoporosis [66]. The abundance of particular miRNA in serum is characteristic of post-menopausal women with a high risk of fracture [67, 68] and in type-2 diabetic women at high fracture risk [67], and these biomarker candidates are currently undergoing validation in independent cohorts.

Several miRNA show promise as biomarkers of ageing in different cells types [69]. For example, miRNA-31-5p has been linked with the ageing phenotype of endothelial cells [70] and may be relevant to ageing bone via cross-talk between the endothelium and osteogenic stem cells [69]. A significant age-related increase in miR-188 was observed in bone-marrow mesenchymal stem cells (BMSCs) of mice, and over-expression of miR-188 resulted in a switch in these cells from osteogenesis to adipogenesis, bone loss, and the accumulation of bone-marrow fat [71]. The role of small non-coding RNA, including miRNA, as potential biomarkers of multiple aspects of health, including musculoskeletal function during ageing, is a very active research area, and there have been a number of independent observations of specific miRNA associated with age-related conditions [72]. For example, Balaskas and colleagues have reported identification of recognised and novel miRNAs, including miR-126, which are changed during cartilage ageing and in OA [73]. However, population cohort studies with a rigorously defined phenotype will be needed to disentangle the effects of ageing on miRNA from those of age-related disease.

## Body composition changes with ageing

Body composition changes during development and ageing, and such changes are linked with changes in function and in the risk of age-related musculoskeletal disease.

### Body-composition assessments

#### Body-composition measurements—recommendations for the toolkit

- Dual-energy X-ray absorptiometry (DXA) remains the most widely recommended method for diagnosing sarcopenia (age-associated loss of muscle mass) as a stand-alone measure or as part of the screening procedure for sarcopenia recommended by the European Working Group on Sarcopenia in Older People (EWGSOP) [74].

#### Body-composition assessments as biomarkers

Body composition changes with ageing as muscle and bone atrophy and, in many cases, there are proportional increases in body fat. This has led to the use of the term sarcopenic obesity. Such compositional changes are linked with changes in function and in the risk of age-related musculoskeletal disease. To date, DXA is the most widely recommended approach for diagnosing sarcopenia based on skeletal muscle mass index (SMI), obtained by dividing appendicular skeletal muscle mass (ASM), evaluated by DXA, by body height squared (SMI = ASM/ht^2^) [75]. According to this definition, individuals presenting an ASM/ht^2^ between 1 and 2 standard deviations (SD) below the gender-specific mean value of young controls are categorised as having class I sarcopenia, while individuals with ASM/ht^2^ >2 SD below this mean have class II sarcopenia (Janssen, Heymsfield *et al.* 2002).

Unfortunately, DXA, like magnetic resonance imaging (MRI) and computed tomography (CT), is expensive and time-consuming, and the required equipment is not portable, thus limiting its use in screening studies. Multi-compartment models that, in addition, involve measures of body volume and total body water, determined by the dilution of labelled water, may be more accurate, but are even more time-consuming. Fat mass and fat-free mass can be estimated by air displacement plethysmography, assuming a constant, and known, density of these body compartments, though significant inter-individual variability exists [76].

Using data from the US National Health and Nutrition Examination Surveys (NHANES), Goodman et al (2013) have developed a practical screening tool to predict low muscle mass, which demonstrates a strong positive association between BMI and SMI [77]. This model could be helpful for use in primary care settings and in treating older populations at risk of sarcopenia. The challenge with this approach is that the ratio of lean to fat tissue declines during ageing (even if body mass and BMI remain unchanged), thus BMI *per se* may be a rather insensitive measure. To address this issue, Prado and colleagues developed sex- and BMI-specific reference curves based on whole-body DXA data, obtained from the 1999-2004 NHANES, to harmonise the classification of body-composition phenotype, which may be particularly useful in the identification of sarcopenic obesity [78].

Bioelectrical impedance analysis (BIA) measures tissue conductivity, which is directly proportional to the amount of electrolyte-rich fluid. Skeletal muscle, the largest tissue in the human body, is electrolyte-rich, and therefore a dominant conductor. BIA has been used to estimate SMI, obtained by dividing appendicular skeletal muscle mass (ASM), evaluated by BIA, by body height squared (ASM/ht^2^), as for the DXA-based approach [75], and type I and type II sarcopenia are defined using the same criteria as for DXA, described above. The assessment of ASM by BIA is inexpensive, easy, and readily reproducible, and is appropriate for both ambulatory and bedridden patients [74].

The BIA skeletal muscle index is strongly correlated to MRI- and DXA-measured skeletal muscle mass [79, 80]. However, specific equations must be used to estimate muscle mass in different populations [81]. Muscle strength is also highly correlated with BIA-measured skeletal muscle mass [82]. However, muscle mass may be overestimated by single-frequency BIA in older people, due to the expansion of extracellular water relative to muscle mass with ageing, although this error is reduced by using multi-frequency BIA [83]. There is evidence that BIA can overestimate the prevalence of sarcopenia [84]. In summary, whilst multi-frequency bioelectrical impedance analysis (BIA) shows promise as a biomarker, this will need further validation.

## Imaging-based assessments of musculoskeletal ageing

*In vivo* imaging techniques including ultrasound, DXA and MRI have been used to measure bone and muscle volume (as a surrogate for mass) and the size of musculoskeletal structures (such as cartilage thickness), and may offer the ability to assess musculoskeletal tissue structure (e.g. with muscle pennation). X-ray techniques such as plain radiography and computed tomography (CT) carry the risk of ionising radiation exposure. DXA involves less ionising radiation exposure, and MRI avoids this risk, though magnet contraindications and the cost and size of equipment limit its application. Ultrasound instruments are highly portable and safe, provide accurate and reliable data, and can be used effectively by non-specialist, but well-trained, staff.

A fundamental problem of identifying markers of musculoskeletal ageing is that it is difficult to distinguish between the effects of ageing *per se* and of disuse. Both factors lead to muscle atrophy, and physical inactivity has an additive effect on the risk of sarcopenia. Physical inactivity is prevalent amongst older people and, e.g. physically active septuagenarians are about 20% less active than their vicenarian counterparts [85]. Moreover, the additive effect of physical inactivity is exacerbated by disease associated with organ failure, and the greater the number of organ failures the greater the atrophy [86].

### Imaging-based assessments of muscle ageing

#### Muscle imaging biomarkers—no current recommendations for the toolkit

- Ultrasound measurement of muscle architecture may enable early detection of changes in muscle mass with ageing, making it a potentially useful next-generation biomarker.
- Extended field of view (EFOV) ultrasound assessment of muscle quality has potential utility in the diagnosis of sarcopenia, and may hold promise as a future biomarker of muscle ageing.

#### Imaging assessments of muscle changes

Whole muscle mass, muscle strength, muscle power and muscle fatigue are all potential markers of muscle changes in ageing [87]. Muscle strength is of particular functional importance [88], while muscle ‘mass’ is a key indicator for sarcopenia.

The introduction and refinement of CT, MRI and DXA has enabled accurate detection of tissue wasting [89]. DXA is the most widely adopted method for the assessment of muscle mass. However, its accuracy varies with size and body fat, as well as age, and results may be biased due to its limited differentiation between water and bone-free lean tissue. Moreover, all three methods are expensive and may be available only at larger institutions.

#### Ultrasound imaging of muscle

Ultrasound imaging is a reliable and sensitive technique for estimating muscle volume in clinical settings, and for detecting changes in muscle mass [90]. Ultrasound waves are reflected by skin, fascia and muscle, and can be used to measure muscle architecture (i.e. thickness, fascicle length, pennation angle and cross-sectional area) in different types of muscles. Ultrasound has been used to assess muscle changes in health and disease, including assessment of changes in muscle architecture with contraction [91], exercise-induced hypertrophy, disuse-atrophy and ageing [92–95]. In addition, ultrasound-derived proxy measures may be useful indices of muscle quality/strength [96].

When assessing hypertrophy or atrophy and sarcopenia, it is useful to establish not only how much but also where muscle is added or lost. This information can be obtained by ultrasound, which can distinguish effects on sarcomeres placed in series (fascicle length) or in parallel (pennation angle and muscle thickness or cross-sectional area). There is preliminary evidence that fascicle length (Lf) is reduced to a smaller extent than muscle thickness (Tm) in sarcopenia, so that the Lf/Tm ratio increases with the severity of sarcopenia; this ratio has been proposed as an ‘Ultrasound Sarcopenia Index (USI)’ [97].

The emerging technique of EFOV ultrasound is comparable to MRI in terms of scope and accuracy. EFOV enables visualisation of the entire vastus lateralis muscle in the plane of scanning, and can be used to study its regional adaptations in response to training [98]. An interesting application of EFOV ultrasound is the assessment of muscle quality as muscle echogenicity (by grey-scale analysis). A recent study concluded that EFOV ultrasound may be used reliably to assess muscle size and quality simultaneously, with high reproducibility, from a single ultrasound scan [99]. Muscle echogenicity is inversely correlated with muscle strength in middle-aged people of both sexes and in elderly men [100, 101]. This may be a potentially useful biomarker of sarcopenia.

### Ultrasound assessment of tendon

#### Tendon ultrasound—no current recommendations for the toolkit

- Biomechanical assessment (tendon stiffness and Young’s modulus) of tendon function measured by ultrasound is a potential future biomarker of ageing in human tendons.

#### Ultrasound studies of tendon

The cross-sectional area of the tendon can be measured with either ultrasound or MRI, and an effect of ageing has been demonstrated in the patellar tendon using ultrasound at a site proximal, but not distal, to tibia insertion within the tendon [102].

Further studies have confirmed the utility of ultrasound in assessing tendon injury and tissue biomechanics. For example, in a study designed to classify rotator cuff tendinopathy in 464 older women (aged 65–87 years), the use of high definition ultrasound revealed a close association between tendon pathology, age and pain [103]. Techniques are being developed to study the sliding of tendon fascicles under load via frame-to-frame speckle tracking [104], and these demonstrate non-uniform deformation patterns, with deep portions of the tendon displacing more than superficial portions, and a reduction in non-uniformity among middle aged versus young adults. Ultrasound assessment can also be used to measure tendon length and force, and to calculate tendon stiffness and strain [105].

Work by Maganaris, Narici and others [106–109] has shown decreased tendon stiffness and Young’s modulus with ageing, assessed by ultrasound, and substantial recovery of tendon stiffness with resistive training [94, 110]. Thus, tendon stiffness and Young’s modulus measured by ultrasound are potential future biomarkers of ageing in human tendons.

### Imaging of joint cartilage

#### Joint imaging—no current recommendations for the toolkit

- A variety of compositional MRI techniques used for the evaluation of cartilage degeneration in OA may hold potential as next-generation biomarkers of ageing, if it can be shown that they change with ageing, independently of OA. T2 imaging is the most widely used of these, while dGEMRIC (delayed gadolinium-enhanced MRI of cartilage) and T1ρ MRI may reflect the proteoglycan content of cartilage. Musculoskeletal ultrasound has some inherent limitations with respect to imaging cartilage, especially since a 90° incident angle is required for good image quality of the cartilage surface. While knee femoral cartilage can be imaged, tibial and most hand joints cannot, especially in those whose flexion is limited by age or pathology. There are no published population cohort data on femoral cartilage thickness, but recent case-control studies [111] and methodological work [112] how that undertaking such measurements in cohort studies is feasible.

#### MRI of joints

The great majority of work in the imaging of joint cartilage has focused on cartilage changes in OA, since there are no markers of cartilage ageing per se. An advantage of MRI is its ability to image cartilage directly. Most commonly, MRI assessment of OA involves one or more of the following: semi-quantitative scoring (SQS) of joint pathology, cartilage volume quantification or compositional MRI.

Compositional MRI techniques for evaluation of cartilage include:

- T2 mapping is straightforward and widely used, and probably provides more information than does structural imaging. Several studies have shown an association with age, and recent work has suggested a weak significant association even without radiographic OA or morphological evidence of cartilage loss on MRI [113].

- Delayed gadolinium-enhanced MRI of cartilage (dGEMRIC) is technically straightforward, but requires intravenous contrast agent and may be challenging in practice. It may be more specific than T2 mapping for proteoglycan content [114].
- Another relaxation parameter, T1ρ, is believed to respond to proteoglycan content, and may be more specific than T2, though its measurement is technically more challenging. There is some suggestion that T1ρ may be better correlated with age than is T2 in healthy volunteers [115].
- Glycosaminoglycan chemical exchange saturation transfer (gagCEST) imaging is technically difficult, and works best at high magnetic field strength. Again, it is thought to be strongly dependent on proteoglycan content; preliminary work suggests that it is promising for detecting cartilage damage, but further studies are needed [114].
- Ultrashort echo-time T2* (UTE-T2*) measurement is a developing technique with the potential to assess deep cartilage [116].

These more advanced techniques promise to have the sensitivity and discriminatory power to detect changes in joint cartilage due to ageing *per se*, independent of OA.

## Functional assessments

Functional assessment tests the workings of the integrated musculoskeletal system. This brings the advantages of direct relevance to clinical state and quality of life, for which system integration is important. A general disadvantage is that the relationships between function and underlying structure and physiology may be complex, particularly in relation to assessing functional capacity in daily life.

### Functional assessment of muscle ageing

#### Functional assessment of muscle—recommendations for the toolkit

- The short physical performance battery (SPPB) of tests, comprising gait speed over 4 m, standing balance, hand grip strength (in both arms) and chair raise test, has been extensively validated. The SPPB should be combined with measurements of lower limb strength.
- The locomotor domain of the NIH toolbox for assessment of neurological and behavioural function represents an alternative approach, combining most of the SPPB tests (hand grip strength, knee extensor isometric strength, walking speed and balance) with endurance and dexterity tests [10].

#### Physical capability testing

The SPPB remains an effective and inexpensive protocol, especially suited to use in those aged over 65–70 years [117]. In a systematic review, the SPPB was the test battery with the highest scores for reliability, validity and responsiveness [118]. In addition, the SPPB has been widely investigated in different populations ranging from vigorous, or independent, to frail.

Hand grip strength shows good correlations with lean body mass and the SMI, and also with leg strength in frail older people. It is advisable to measure hand grip in both arms, since significant differences between the dominant and the non-dominant side have been observed for hand grip strength, in both sexes, but not for lower limb strength [119]. However, hand grip strength does not provide a valid means of evaluating the efficacy of intervention programs to increase muscle mass or function in an older population [120], and is likely to be less sensitive to training or rehabilitation interventions than lower limb strength [120]. Therefore, lower limb strength should be incorporated as a key indicator of musculoskeletal function. The assessment of lower limb strength should include the maximal voluntary isometric contraction (MVIC) of both knee extensor and flexor muscle groups, enabling the simultaneous evaluation of the rate of torque development (RTD). RTD of knee flexors (KF) correlates with the number of falls in older people [121], an important consideration given the increasing prevalence of falls with ageing. Whilst muscle power (the product of force and velocity of contraction) is more affected by ageing than is muscle strength [122], there is no practicable and inexpensive set of tests for measurement of muscle power. Therefore, RTD could act as a surrogate for muscle power, providing important information about an individual’s ability to develop force dynamically, since it is correlated with MVIC, muscle fibre characteristics, tendon stiffness and neural activation capability [123, 124].

The use of generic cut-off threshold values for muscle strength (e.g. hand grip) and function (e.g. gait speed) to pre-screen subjects at potential risk of sarcopenia [74] may overestimate risk compared with the use of specific cut-off values from the population under study [125], and the latter approach has been recommended for individual research studies and for population screenings [125]. Indeed, ageing can affect body composition and physical performance in older adults very differently depending on lifestyle, culture and ethnicity [126, 127]. There are also gender differences in rate of decline in muscle strength and mass, with faster rates of decline in men [128].

Finally, we recommend the locomotor domain of the NIH toolbox for assessment of neurological and behavioural function [10] since it encompasses measures that are feasible, valid, and inexpensive. It comprises five sub-domains of motor function: dexterity, endurance, locomotion, balance and strength, including several of the tests discussed above, such as hand grip strength, knee extensor isometric strength, walking speed, and balance, together with a 2-min endurance walking test (a modified version of the 6-min walking test) and a dexterity text (the Rolyan 9-hole pegboard). A potential limitation of this toolbox is that the sample size used for its validation was small. In addition, its use by non-expert users (as envisioned by the proposers) could be critical, especially for tests such as the use of a hand-held dynamometer for the assessment of lower limb strength. However, hand-held dynamometry has the advantage of portability and reduced cost compared with traditional laboratory-based lower limb dynamometry.

### Functional assessment of tendon ageing

#### Functional assessment of tendon—no current recommendations for the toolkit

Self-assessment questionnaires and similar instruments can be used to determine disability and tendon pain, but not loss of function. However, such instruments are not sensitive to ageing per se, so cannot be recommended for the toolkit.

#### Assessment of tendon ageing

Tendons are formed of highly aligned collagenous tissue, consisting predominantly of type I collagen, and the risk of tendon injury increases with age. Rotator cuff injury, for example, has a prevalence of 9.7% in individuals under 20 years of age, and 62% in those over 80 years [129].

Self-assessment questionnaires and similar instruments can determine disability and pain associated with tendinopathy reliably but, in general, they are not sensitive to ageing *per se* and so cannot be recommended for the toolkit. A further issue is that many such instruments, e.g. for the assessment of Achilles tendinopathy, are sports-related rather than age-related [130]. Other questionnaires are available for assessing shoulder pain and disability [131], and all have the advantage of being simple to use and low cost.

### Assessment of balance control in relation to ageing

#### Balance control—no current recommendations for the toolkit

- While valid and reliable tests of balance are available, and have been shown to be associated with muscle function, balance control is affected by factors other than musculoskeletal ageing *per se*. In addition, it is unclear whether balance problems precede decreases in muscle function. Hence, the suitability of balance assessment as a biomarker of musculoskeletal ageing is not supported by the available evidence.
- Tests of reactive balance control are more demanding and, consequently, are more likely to reflect early changes in muscle function, and thus show promise as future biomarkers of musculoskeletal ageing.

#### Balance assessment

Balance can be defined as the ability to control the position of the body’s centre of mass in relation to the base of support. Balance control is an important determinant of falls risk, mobility and independence. It is dependent on functioning of muscle-tendon units in the trunk and lower extremities, but also on sensory information from multiple modalities, brain function (including function of pre-frontal areas), and regulation of blood pressure. Consequently, balance assessment is highly sensitive to subtle changes in health status. To a large extent, balance control is context dependent, and thus assessment of balance may need multiple tests.

A history of falls remains the strongest predictor of future falls [132] and is an important marker of balance problems, and so falls-related questionnaires can provide insight into balance control. Several instruments measure the fear of falling and its converse, balance confidence. The international Falls Efficacy Scale (FES-i) comprises either 7 or 16 items that measure fear of falling, i.e. the concern that one may fall during daily activities, and this has been shown to be associated with balance impairments [133]. In contrast, the Activities-specific Balance Confidence (ABC) scale comprises 16 items that assess balance confidence [134]. Both questionnaires have been widely used in groups without significant cognitive disorders.

Static balance tests can be used to measure, for example, the ability to maintain balance for 10 s in various standing conditions (e.g. with legs side by side, tandem, semi-tandem, on one leg, with eyes open or closed). Static balance tests can be further augmented by measuring postural sway, assessed as movement of the centre of pressure (CoP) or amplitude/speed of the centre of mass (CoM), which is the gold standard [135].

Dynamic balance tests typically involve external perturbations of the standing balance, as in the limits of stability (LOS) [136] and mediolateral balance assessments (MELBA) [137] tests. In the LOS test, subjects are asked to lean in different directions (forwards, backwards and sideways) as far as possible, while in MELBA, subjects are asked to track a sideways moving target with their CoM by shifting their weight sideways. Other dynamic balance tests assess reactive control, for example to platform translations and rotations, via stepping responses or time to stabilisation.

Balance control in daily life activities such as walking poses challenges in addition to those encountered in the tests described above. Functional balance tests, such as the Performance Oriented Mobility Assessment (POMA), Berg Balance Scale (BBS), Timed Up and Go (TUG) test, and the SPPB balance elements, focus on daily-life activities that challenge balance control, and so may be more appropriate than the more ‘artificial’ tests of static and dynamic balance. These tests also have the advantages of being simple and cheap, and show good reliability between observers. However, they have ceiling effects and they are not very specific for balance. Assessment of treadmill walking may have potential to assess balance in a realistic task, since its outcomes in terms of variability and local dynamic stability correlate with fall history [138]. Two further tests are worthy of consideration as future biomarkers: narrow beam walking [139], which has the advantage of simplicity, and responsiveness to virtual obstacles or stepping stones projected onto a treadmill or onto the floor to form an interactive walkway [140, 141].

Assessment of indicators of balance control during activities of daily life may provide a complete picture of the quality of balance control in a relevant context at relatively low cost. Measured over a week, such parameters provide a fairly good prediction of future falls [132], but independent validation of these findings is necessary.

Whilst clearly relevant to risk of falling, the relevance of these measures to musculoskeletal ageing *per se* remains to be determined. Most of the balance tests described above are associated with muscle function. For example, fear of falling tested with a single-item questionnaire is correlated with knee extensor strength [142], the time that older adults can stand on one leg is correlated with muscle strength, [143, 144], postural sway is correlated with the strength, and cross-sectional area, of trunk muscles [145], and local dynamic stability of walking is correlated with knee extensor strength [142]. For dynamic tests, results on the MELBA test are correlated with the strength and position sense of the hip abductor muscles [146], and the ability to regain balance within one step after a platform perturbation is correlated with the muscle strength of ankle and trunk muscles [147, 148]. Finally, older adults with weak and slow muscles are successful much less often in regaining their balance after tripping over an obstacle than their stronger peers [149]. However, most of these associations are found in adults aged over 65 years, and it is unsure whether such associations exist earlier in life. While it seems likely that this may be the case for the more challenging balance tests, this remains to be demonstrated.

### Real-life monitoring of musculoskeletal function

#### Real-life monitoring—no current recommendations for the toolkit

There is no fully validated system for real-life monitoring of musculoskeletal function to date. Robust accuracy and validity metrics have been reported for some features, but engineering challenges remain and definitions, protocols and outcomes need to be standardised.

#### Advantages and challenges of real-life monitoring

The potential advantage of real-life monitoring is that it may provide an accurate picture of people’s day-to-day activity in their usual environment, in contrast to laboratory testing which is necessarily contrived. This is exemplified by global studies that demonstrate inequality in patterns of physical activity across age, gender, BMI, ethnicity and geography [150]. However, it is important to distinguish between what people do in ‘real life’ and what they are capable of doing. In terms of measuring musculoskeletal ageing, it may more important to measure ‘capacity’ or ‘capability’ than what people actually do, since the latter is largely a reflection of an ‘unchallenged’, habitual and largely sedentary lifestyle.

Real-life monitoring presents technical challenges. For example, it may involve a variety of wearable technology and connected devices (WTCD), from simple triaxial accelerometers to gyroscopes and magnetometers, allowing continuous detection of postures, postural transitions and activities. While the utility, reliability and validity of gait measurement with traditional gold standard laboratory-based systems (e.g. three-dimensional motion capture systems and instrumented mattresses, such as GAITRite) is broadly accepted, the performance characteristics of novel WTCD are often not reported formally, or reported inconsistently using a variety of protocols and measures [151]. Efforts are currently underway to redress these deficits [152–154].

The approach of using generic low-cost (<£100) body-worn movement monitors, analysed using published algorithms, may ultimately offer an affordable and scalable solution for quantitative gait evaluation in both multicentre studies and real-world settings. Nevertheless, algorithm development presents considerable technical challenges, so this approach is not yet ready for recommendation in the musculoskeletal ageing assessment toolkit.

## Conclusions, future perspectives and priorities for research

In this report, we have reviewed potential biomarkers of musculoskeletal ageing, and the key biological and technical issues that bear on their inclusion or otherwise in a CIMA toolkit. Our recommendations are summarised in Table 1.

**Table 1.** CIMA- recommended biomarkers of musculoskeletal ageing in humans

Biomarker	Component of musculoskeletal system assessed
PINP and CTX	Biomarkers of bone turnover
Serum creatinine	Biomarker of skeletal muscle mass
DXA	Assessment of body composition
SPPB or Locomotion domain of NIH Toolbox	Assessment of physical capability

Overall progress in the field of developing and validating biomarkers of musculoskeletal ageing in humans has been slow and uneven and, to date, there are relatively few accepted and reliable biomarkers for ageing of this major body system. For bones and muscle, we have some useful biomarkers of ageing, but for the other components of the musculoskeletal system—tendons and joints—no such biomarkers are currently available. There are well-established markers of body composition, e.g. DXA and of physical capability—we recommend the SPPB or the Locomotion domain of the NIH Toolbox—but changes in both domains are not necessarily specific consequences of musculoskeletal ageing. However, measures of physical capability have the advantage that, to a considerable extent, they reflect function of the integrated musculoskeletal system.

The slow and uneven progress in developing biomarkers of musculoskeletal ageing parallels the situation with biomarkers of ageing *per se* [5, 6, 155], and reflects both the complexity and heterogeneity of ageing, and the difficulties in distinguishing between biomarkers of an ageing body system and biomarkers of age-related disease in that system. This review reveals that much research effort has been devoted to disease-related biomarkers, and relatively little to biomarkers of musculoskeletal ageing itself. There are both conceptual and practical reasons for this imbalance in research focus, and the problems are amplified at older ages when multi-morbidity and polypharmacy are more common [156]. This suggests that research on biomarkers of musculoskeletal ageing is likely to be more rewarding if it is conducted earlier in the ageing trajectory.

Despite the difficulties in defining and measuring ageing, there has been significant progress in recent years in understanding its biological basis, and this may offer new insights and directions for the development of biomarkers. For example, Lopez-Otin *et al.* identified nine hallmarks of ageing which are common to many, perhaps all, multicellular species that exhibit ageing [157]. One of these hallmarks is epigenetic alterations, and it is now clear that, at some genomic sites, DNA methylation status changes with age in a predictable manner. Whilst a number of ‘DNA methylation clocks’ have been proposed, that by Horvath appears to be among the most useful [158]. In Horvath’s model, DNA methylation at 353 specific CpG sites shows considerable inter-individual differences between predicted and chronological age, and this difference between ‘methylation age’ and chronological age (Δ_age_) is predictive of all-cause mortality in later life [159].

‘Methylation age’ has utility in assessing ageing of multiple tissues but, as yet, there is limited information about its value for musculoskeletal tissues. Using data from Ribel-Mason and colleagues [160], Horvath observed that in muscle, DNA methylation age correlated poorly with chronological age [158]. In OA patients, accelerated epigenetic ageing was demonstrated in articular cartilage but not in bone or blood [161]. We anticipate further developments in this area as approaches for genome-wide analysis of DNA methylation are applied to ageing cohorts in which there are relevant additional measures of musculoskeletal function. In the meantime, other epigenetic-based biomarkers of musculoskeletal ageing are emerging, particularly those based on non-coding RNA species [162]. In this review, we have summarised progress in identification of miRNA which are linked with risk of osteoporosis, and which may be biomarkers of joint ageing more generally. Recently, differential expression of a number of snoRNA (another group of non-coding, regulatory RNA) in young versus old and normal versus OA murine joints and serum has been described, which suggests that snoRNA are also putative markers of musculoskeletal ageing [61]. In addition, a wide range of biological approaches, including proteomics and metabolomics [163], are being used to identify and validate biomarkers of ageing [164], some of which may be applicable to the musculoskeletal system.

This review shows that there are few reliable biomarkers of musculoskeletal ageing in humans. This is a major evidence gap which limits research on the processes involved in, and factors modulating, musculoskeletal ageing. It is also a major impediment to the design and conduct of intervention studies that aim to maintain good musculoskeletal function during ageing. Further, few biomarker approaches (excepting those that attempt to monitor physical function in real-life situations) consider all of the musculoskeletal tissues as an integrated system, and there are gaps in biomarkers for assessing joint and tendon function during ageing. Identification of these research gaps should stimulate funders and researchers to address these issues, not least because of the very substantial contribution that poor musculoskeletal ageing makes to the burden of age-related frailty and disability [165]. Since debilitating age-related disorders of the musculoskeletal system have major adverse effects on the independence and quality of life of older individuals which, by limiting physical activity, amplify age-related risks of multiple cardio-metabolic diseases, major cancers and neurodegenerative diseases [166, 167], this underscores the imperative to assess and maintain good musculoskeletal function during ageing.

Success in the search for reliable biomarkers of musculoskeletal ageing will require innovation, not only in the application of new technologies and emerging understanding of the biology of the ageing process, but also in experimental design. There may be merit in the study of individual trajectories in musculoskeletal function during middle age, in advance of the disability and disease (including non-musculoskeletal diseases) that are likely to be major confounders. This will require repeated measures of musculoskeletal function, appropriate imaging and collection of biological samples for biomarker assessment at more frequent intervals than is usual in large ageing cohorts. In addition, measurement tools will need to be much more sensitive to detect the subtler age-related changes that are characteristic of the ageing phenotype. In summary, progress in developing and validating biomarkers of musculoskeletal ageing will require multi-disciplinary teams that are willing to embrace new ways of working. Embedding studies within large population cohorts of initially relatively healthy individuals, such as the UK Biobank, innovation in measurement science including participant self-monitoring, and the opportunities that arise from interrogation of ‘big data’ obtained from linkage to routinely-collected data on healthcare use may all be helpful in achieving this goal.

In conclusion, this report represents the first systematic attempt to develop a toolkit suitable for assessing musculoskeletal ageing. Reviews by an expert, multi-disciplinary group have revealed remarkably few biomarkers of musculoskeletal ageing in humans which are suitable for characterising and quantifying musculoskeletal function during ‘normal’ ageing, or which would be appropriate for use as outcome measures in intervention studies designed to enhance healthy musculoskeletal ageing (Table 1). Given that the musculoskeletal system functions in an integrated way, measures of physical capability, e.g. the SPPB or the Locomotion domain of the NIH Toolbox, may have particular utility in assessing musculoskeletal ageing because, to a considerable extent, these tests reflect function of the whole system. There appear to be no reliable measures of ageing of joints and tendons, and those markers available for bone and muscle assess only part of the loss of quantity and quality experienced during ageing. There is a high priority to address this research gap, and we have suggested some conceptual and practical approaches that may provide traction in this difficult, but important, area of public health.

A summary of the full names of acronyms used in this report is included in Table 2.

**Key points**

- Biochemical biomarkers: We considered a wide range of biochemical markers and two well-established serum markers of bone turnover, i.e. N-terminal propeptide of type I procollagen and C-terminal cross-linked telopeptide of collagen type I were recommended for the toolkit. ‘Omics’ approaches are revealing potential new candidate biomarkers and non-coding RNA species detectable in the circulation show particular promise as future biomarkers.
- Body-composition biomarkers: Body composition changes during development and ageing, and such changes are linked with changes in function and in the risk of age-related musculoskeletal disease. We recommended serum creatinine (with appropriate dietary control) as a biomarker of skeletal muscle mass and dual-energy X-ray absorptiometry for diagnosing sarcopenia (age-associated loss of muscle mass).
- Imaging assessments: Multiple *in vivo* imaging techniques were considered including ultrasound, dual-energy X-ray absorptiometry and magnetic resonance imaging. Some of these are well-established for assessment/ diagnosis of musculoskeletal disease but none were considered suitable, as yet, for measuring musculoskeletal ageing. Ultrasound measurement of muscle architecture may enable early detection of changes in muscle mass with ageing, making it a potentially useful next-generation biomarker.
- Functional assessments: Functional assessments have the advantage of direct relevance to clinical state and quality of life, for which system integration is important. The Short physical performance battery or the Locomotion domain of NIH Toolbox are recommended.

**Table 2.** Explanation of acronyms used in report

Acronym	Full name
3MH	3-Methylhistidine
ABC	Activities-specific Balance Confidence
ASM	Appendicular skeletal muscle mass
BBS	Berg Balance Scale
BIA	Bioelectrical impedance analysis
BMI	Body Mass Index
BMSCs	Bone-marrow mesenchymal stem cells
C6M	Matrix MMP-generated degradation fragment of collagen 6
CAFs	C-terminal agrin fragments
CIMA	MRC—Arthritis Research UK Centre for Integrated research into Musculoskeletal Ageing
CoM	Centre of mass
COMP	Cartilage oligomeric matrix protein
CoP	Centre of pressure
CT	Computed tomography
CTX, CTX-I, CTX-II	Telopeptide of Type I Collagen
dGEMRIC	Delayed gadolinium-enhanced MRI of cartilage
DPD	Deoxypyridinoline
DXA	Dual-energy X-ray absorptiometry
EFOV	Extended field of view
EWGSOP	European Working Group on Sarcopenia in Older People
FES-i	International Falls Efficacy Scale
gagCEST	Glycosaminoglycan chemical exchange saturation transfer
GDF	Growth Differentiation Factor
GDF8	Growth Differentiation Factor 8 (Myostatin)
GFR	Glomerular filtration rate
HAP	Healthy ageing phenotype
IC6	Type VI collagen N-terminal globular domain epitope
ICTP	Carboxy-terminal cross-linked telopeptide of type I collagen generated by matrix metalloproteinases
κB	Kappa-Β
KF	Knee flexors
Lf	Fascicle length
LOS	Limits of stability
MALDI-MSI	Matrix-assisted laser desorption ionisation mass spectrometry imaging
MELBA	Mediolateral balance assessments
MMP	Matrix metalloproteinase
MRI	Magnetic resonance imaging
MVIC	Maximal voluntary isometric contraction
NHANES	US National Health and Nutrition Examination Surveys
NMJ	Neuromuscular junction
NTX	N-terminal cross-linked telopeptide of collagen type I
OA	Osteoarthritis
P3NP	N-terminal peptide of procollagen type III
PICP, PINP	C- and N-terminal pro-peptides of type I procollagen
PINP	Propeptide of type I procollagen
POMA	Performance Oriented Mobility Assessment
RANKL	Receptor activator of nuclear factor κB
RNA	Ribonucleic acid
RTD	Rate of torque development
SMI	Skeletal muscle mass index
snoRNAs	Small nucleolar RNAs
SPPB	Short physical performance battery
SQS	Semi-quantitative scoring
Tm	Muscle thickness
TRACP5b, also known as TRACP5b	Tartrate-resistant acid phosphatase isoform 5b
TUG	Timed Up and Go test
USI	Ultrasound Sarcopenia Index
UTE-T2*	Ultrashort echo-time T2*
WTCD	Wearable technology and connected devices

## Footnotes

1
http://www.cimauk.org/

## References

[1] Kirkwood TB Understanding the odd science of aging. Cell 2005; 120: 437–47. 15734677 10.1016/j.cell.2005.01.027

[2] Belsky DW, Caspi A, Houts R, Cohen HJ, Corcoran DL, Danese A et al Quantification of biological aging in young adults. Proc Natl Acad Sci USA 2015; 112: E4104–10. 26150497 10.1073/pnas.1506264112 PMC4522793

[3] Butler RN, Sprott R, Warner H, Bland J, Feuers R, Forster M et al Biomarkers of aging: from primitive organisms to humans. J Gerontol A Biol Sci Med Sci 2004; 59: B560–7. 15215265 10.1093/gerona/59.6.b560

[4] Franco OH, Karnik K, Osborne G, Ordovas JM, Catt M, van der Ouderaa F Changing course in ageing research: The healthy ageing phenotype. Maturitas 2009; 63: 13–9. 19282116 10.1016/j.maturitas.2009.02.006

[5] Lara J, Godfrey A, Evans E, Heaven B, Brown LJ, Barron E et al Towards measurement of the Healthy Ageing Phenotype in lifestyle-based intervention studies. Maturitas 2013; 76: 189–99. 23932426 10.1016/j.maturitas.2013.07.007

[6] Lara J, Cooper R, Nissan J, Ginty AT, Khaw KT, Deary IJ et al A proposed panel of biomarkers of healthy ageing. BMC Med 2015; 13: 222. 26373927 10.1186/s12916-015-0470-9 PMC4572626

[7] Tampubolon G Trajectories of the healthy ageing phenotype among middle-aged and older Britons, 2004–2013. Maturitas 2016; 88: 9–15. 27105690 10.1016/j.maturitas.2016.03.002 PMC4850932

[8] Sebastiani P, Thyagarajan B, Sun F, Schupf N, Newman AB, Montano M et al Biomarker signatures of aging. Aging Cell 2017; 16: 329–38. 28058805 10.1111/acel.12557 PMC5334528

[9] Collino S, Martin FP, Karagounis LG, Horcajada MN, Moco S, Franceschi C et al Musculoskeletal system in the old age and the demand for healthy ageing biomarkers. Mech Ageing Dev 2013; 134: 541–7. 24269882 10.1016/j.mad.2013.11.003

[10] Reuben DB, Magasi S, McCreath HE, Bohannon RW, Wang YC, Bubela DJ et al Motor assessment using the NIH Toolbox. Neurology 2013; 80: S65–75. 23479547 10.1212/WNL.0b013e3182872e01 PMC3662336

[11] Engelfriet PM, Jansen EH, Picavet HS, Dolle ME Biochemical markers of aging for longitudinal studies in humans. Epidemiol Rev 2013; 35: 132–51. 23382477 10.1093/epirev/mxs011 PMC4707878

[12] Naylor KE, Jacques RM, Paggiosi M, Gossiel F, Peel NF, McCloskey EV et al Response of bone turnover markers to three oral bisphosphonate therapies in postmenopausal osteoporosis: the TRIO study. Osteoporos Int 2016; 27: 21–31. 25990354 10.1007/s00198-015-3145-7

[13] Fink E, Cormier C, Steinmetz P, Kindermans C, Le Bouc Y, Souberbielle JC Differences in the capacity of several biochemical bone markers to assess high bone turnover in early menopause and response to alendronate therapy. Osteoporos Int 2000; 11: 295–303. 10928218 10.1007/PL00004183

[14] Hannon R, Blumsohn A, Naylor K, Eastell R Response of biochemical markers of bone turnover to hormone replacement therapy: impact of biological variability. J Bone Miner Res 1998; 13: 1124–33. 9661076 10.1359/jbmr.1998.13.7.1124

[15] Rogers A, Glover SJ, Eastell R A randomised, double-blinded, placebo-controlled, trial to determine the individual response in bone turnover markers to lasofoxifene therapy. Bone 2009; 45: 1044–52. 19665601 10.1016/j.bone.2009.07.089

[16] Vasikaran S, Eastell R, Bruyere O, Foldes AJ, Garnero P, Griesmacher A et al Markers of bone turnover for the prediction of fracture risk and monitoring of osteoporosis treatment: a need for international reference standards. Osteoporos Int 2011; 22: 391–420. 21184054 10.1007/s00198-010-1501-1

[17] Eastell R, Nagase S, Small M, Boonen S, Spector T, Ohyama M et al Effect of ONO-5334 on bone mineral density and biochemical markers of bone turnover in postmenopausal osteoporosis: 2-year results from the OCEAN study. J Bone Miner Res 2014; 29: 458–66. 23873670 10.1002/jbmr.2047

[18] Chavassieux P, Portero-Muzy N, Roux JP, Garnero P, Chapurlat R Are biochemical markers of bone turnover representative of bone histomorphometry in 370 postmenopausal women? J Clin Endocrinol Metab 2015; 100: 4662–8. 26505821 10.1210/jc.2015-2957

[19] Eastell R, Colwell A, Hampton L, Reeve J Biochemical markers of bone resorption compared with estimates of bone resorption from radiotracer kinetic studies in osteoporosis. J Bone Miner Res 1997; 12: 59–65. 9240726 10.1359/jbmr.1997.12.1.59

[20] Asamiya Y, Tsuchiya K, Nitta K Role of sclerostin in the pathogenesis of chronic kidney disease-mineral bone disorder. Ren Replace Ther 2016; 2: 1–8. [journal article].

[21] Voorzanger-Rousselot N, Journe F, Doriath V, Body JJ, Garnero P Assessment of circulating Dickkopf-1 with a new two-site immunoassay in healthy subjects and women with breast cancer and bone metastases. Calcif Tissue Int 2009; 84: 348–54. 19252761 10.1007/s00223-009-9225-y

[22] Bonnet N, Garnero P, Ferrari S Periostin action in bone. Mol Cell Endocrinol 2016; 432: 75–82. 26721738 10.1016/j.mce.2015.12.014

[23] Lewiecki EM New targets for intervention in the treatment of postmenopausal osteoporosis. Nature Reviews. Rheumatology 2011; 7: 631–8. 21931340 10.1038/nrrheum.2011.130

[24] Rousseau JC, Delmas PD Biological markers in osteoarthritis. Nat Clin Pract Rheumatol 2007; 3: 346–56. 17538566 10.1038/ncprheum0508

[25] Valdes AM, Meulenbelt I, Chassaing E, Arden NK, Bierma-Zeinstra S, Hart D et al Large scale meta-analysis of urinary C-terminal telopeptide, serum cartilage oligomeric protein and matrix metalloprotease degraded type II collagen and their role in prevalence, incidence and progression of osteoarthritis. Osteoarthritis Cartilage 2014; 22: 683–9. 24576742 10.1016/j.joca.2014.02.007

[26] De Ceuninck F, Sabatini M, Pastoureau P Recent progress toward biomarker identification in osteoarthritis. Drug Discov Today 2011; 16: 443–9. 21262380 10.1016/j.drudis.2011.01.004

[27] Kraus VB Osteoarthritis year 2010 in review: biochemical markers. Osteoarthritis Cartilage 2011; 4: 346–53. 10.1016/j.joca.2011.02.002 PMC3065527 21320614

[28] Hosnijeh FS, Runhaar J, van Meurs JB, Bierma-Zeinstra SM Biomarkers for osteoarthritis: can they be used for risk assessment? A systematic review. Maturitas 2015; 82: 36–49. 25963100 10.1016/j.maturitas.2015.04.004

[29] Rousseau J, Garnero P Biological markers in osteoarthritis. Bone 2012; 51: 265–77. 22538364 10.1016/j.bone.2012.04.001

[30] Van Spil WE, Welsing PM, Bierma-Zeinstra SM, Bijlsma JW, Roorda LD, Cats HA et al The ability of systemic biochemical markers to reflect presence, incidence, and progression of early-stage radiographic knee and hip osteoarthritis: data from CHECK. Osteoarthritis Cartilage 2015; 23: 1388–97. 25819579 10.1016/j.joca.2015.03.023

[31] Verma P, Dalal K Serum cartilage oligomeric matrix protein (COMP) in knee osteoarthritis: a novel diagnostic and prognostic biomarker. J Orthop Res 2013; 31: 999–1006. 23423905 10.1002/jor.22324

[32] Kluzek S, Bay-Jensen AC, Judge A, Karsdal MA, Shorthose M, Spector T et al Serum cartilage oligomeric matrix protein and development of radiographic and painful knee osteoarthritis. A community-based cohort of middle-aged women. Biomarkers 2015; 20: 557–64. 26848781 10.3109/1354750X.2015.1105498 PMC4819573

[33] Bay-Jensen AC, Andersen TL, Charni-Ben Tabassi N, Kristensen PW, Kjaersgaard-Andersen P, Sandell L et al Biochemical markers of type II collagen breakdown and synthesis are positioned at specific sites in human osteoarthritic knee cartilage. Osteoarthritis Cartilage. [Osteoarthritis Cartilage] 2008; 16: 615–23. 10.1016/j.joca.2007.09.006 17950629

[34] de Klerk B, Lafeber FP, van Spil WE Associations of CTX-II with biochemical markers of bone turnover raise questions about its tissue origin: new insights from CHECK. Ann Rheum Dis 2014; 73: e39. 24695012 10.1136/annrheumdis-2014-205494

[35] Garnero P, Sornay-Rendu E, Arlot M, Christiansen C, Delmas PD Association between spine disc degeneration and type II collagen degradation in postmenopausal women: the OFELY study. Arthritis Rheumatol 2004; 50: 3137–44. 10.1002/art.20493 15476251

[36] Garnero P, Ayral X, Rousseau JC, Christgau S, Sandell LJ, Dougados M et al Uncoupling of type II collagen synthesis and degradation predicts progression of joint damage in patients with knee osteoarthritis. Arthritis Rheumatol 2002; 46: 2613–24. 10.1002/art.10576 12384919

[37] Kraus VB, Collins JE, Hargrove D, Losina E, Nevitt M, Katz JN et al Predictive validity of biochemical biomarkers in knee osteoarthritis: data from the FNIH OA Biomarkers Consortium. Ann Rheum Dis 2017; 76: 186–95. 27296323 10.1136/annrheumdis-2016-209252 PMC5851287

[38] Mouritzen U, Christgau S, Lehmann HJ, Tanko LB, Christiansen C Cartilage turnover assessed with a newly developed assay measuring collagen type II degradation products: influence of age, sex, menopause, hormone replacement therapy, and body mass index. Ann Rheum Dis 2003; 62: 332–6. 12634232 10.1136/ard.62.4.332 PMC1754496

[39] Kraus VB, Hargrove DE, Hunter DJ, Renner JB, Jordan JM Establishment of reference intervals for osteoarthritis-related soluble biomarkers: the FNIH/OARSI OA Biomarkers Consortium. Ann Rheum Dis 2017; 76: 179–85. 27343253 10.1136/annrheumdis-2016-209253

[40] Sivan SS, Tsitron E, Wachtel E, Roughley P, Sakkee N, van der Ham F et al Age-related accumulation of pentosidine in aggrecan and collagen from normal and degenerate human intervertebral discs. Biochem J 2006; 399: 29–35. 16787390 10.1042/BJ20060579 PMC1570172

[41] Peffers MJ, Cillero-Pastor B, Eijkel GB, Clegg PD, Heeren RM Matrix assisted laser desorption ionization mass spectrometry imaging identifies markers of ageing and osteoarthritic cartilage. Arthritis Res Ther 2014; 16: R110. 24886698 10.1186/ar4560 PMC4095688

[42] Schiaffino S, Dyar KA, Ciciliot S, Blaauw B, Sandri M Mechanisms regulating skeletal muscle growth and atrophy. FEBS J 2013; 280: 4294–314. 23517348 10.1111/febs.12253

[43] Cesari M, Fielding RA, Pahor M, Goodpaster B, Hellerstein M, van Kan GA et al Biomarkers of sarcopenia in clinical trials-recommendations from the International Working Group on Sarcopenia. J Cachexia Sarcopenia Muscle 2012; 3: 181–90. 22865205 10.1007/s13539-012-0078-2 PMC3424187

[44] Drescher C, Konishi M, Ebner N, Springer J Loss of muscle mass: current developments in cachexia and sarcopenia focused on biomarkers and treatment. J Cachexia Sarcopenia Muscle 2015; 6: 303–11. 26676067 10.1002/jcsm.12082 PMC4670737

[45] Kalinkovich A, Livshits G Sarcopenia—the search for emerging biomarkers. Ageing Res Rev 2015; 22: 58–71. 25962896 10.1016/j.arr.2015.05.001

[46] Patel SS, Molnar MZ, Tayek JA, Ix JH, Noori N, Benner D et al Serum creatinine as a marker of muscle mass in chronic kidney disease: results of a cross-sectional study and review of literature. J Cachexia Sarcopenia Muscle 2013; 4: 19–29. 22777757 10.1007/s13539-012-0079-1 PMC3581614

[47] Epstein M Aging and the kidney. J Am Soc Nephrol 1996; 7: 1106–22. 8866401 10.1681/ASN.V781106

[48] Clark RV, Walker AC, O’Connor-Semmes RL, Leonard MS, Miller RR, Stimpson SA et al Total body skeletal muscle mass: estimation by creatine (*methyl*-d_3_) dilution in humans. J Appl Physiol 2014; 116: 1605–13. 24764133 10.1152/japplphysiol.00045.2014 PMC4064374

[49] Wilkinson DJ, Franchi MV, Brook MS, Narici MV, Williams JP, Mitchell WK et al A validation of the application of D_2_O stable isotope tracer techniques for monitoring day-to-day changes in muscle protein subfraction synthesis in humans. Am J Physiol Endocrinol Metab 2014; 306: E571–9. 24381002 10.1152/ajpendo.00650.2013 PMC3948971

[50] Elia M, Carter A, Bacon S, Smith R The effect of 3-methylhistidine in food on its urinary excretion in man. Clin Sci 1980; 59: 509–11. 7438717 10.1042/cs0590509

[51] Sheffield-Moore M, Dillon EL, Randolph KM, Casperson SL, White GR, Jennings K et al Isotopic decay of urinary or plasma 3-methylhistidine as a potential biomarker of pathologic skeletal muscle loss. J Cachexia Sarcopenia Muscle 2014; 5: 19–25. 24009031 10.1007/s13539-013-0117-7 PMC3953321

[52] Nedergaard A, Sun S, Karsdal MA, Henriksen K, Kjaer M, Lou Y et al Type VI collagen turnover-related peptides-novel serological biomarkers of muscle mass and anabolic response to loading in young men. J Cachexia Sarcopenia Muscle 2013; 4: 267–75. 23943593 10.1007/s13539-013-0114-x PMC3830008

[53] Fragala MS, Jajtner AR, Beyer KS, Townsend JR, Emerson NS, Scanlon TC et al Biomarkers of muscle quality: N-terminal propeptide of type III procollagen and C-terminal agrin fragment responses to resistance exercise training in older adults. J Cachexia Sarcopenia Muscle 2014; 5: 139–48. 24197815 10.1007/s13539-013-0120-z PMC4053565

[54] Reif R, Sales S, Hettwer S, Dreier B, Gisler C, Wolfel J et al Specific cleavage of agrin by neurotrypsin, a synaptic protease linked to mental retardation. FASEB J 2007; 21: 3468–78. 17586728 10.1096/fj.07-8800com

[55] Steubl D, Roos M, Hettwer S, Satanovskij R, Tholen S, Wen M et al Plasma total C-terminal agrin fragment (tCAF) as a marker for kidney function in patients with chronic kidney disease. Clin Chem Lab Med 2016; 54: 1487–95. 26876812 10.1515/cclm-2015-1027

[56] Steinbeck L, Ebner N, Valentova M, Bekfani T, Elsner S, Dahinden P et al Detection of muscle wasting in patients with chronic heart failure using C-terminal agrin fragment: results from the Studies Investigating Co-morbidities Aggravating Heart Failure (SICA-HF). Eur J Heart Fail 2015; 17: 1283–93. 26449626 10.1002/ejhf.400

[57] Landi F, Calvani R, Lorenzi M, Martone AM, Tosato M, Drey M et al Serum levels of C-terminal agrin fragment (CAF) are associated with sarcopenia in older multimorbid community-dwellers: results from the ilSIRENTE study. Exp Gerontol 2016; 79: 31–6. 27015736 10.1016/j.exger.2016.03.012

[58] Schafer MJ, Atkinson EJ, Vanderboom PM, Kotajarvi B, White TA, Moore MM et al Quantification of GDF11 and myostatin in human aging and cardiovascular disease. Cell Metab 2016; 23: 1207–15. 27304512 10.1016/j.cmet.2016.05.023 PMC4913514

[59] Fujita Y, Taniguchi Y, Shinkai S, Tanaka M, Ito M Secreted growth differentiation factor 15 as a potential biomarker for mitochondrial dysfunctions in aging and age-related disorders. Geriatr Gerontol Int 2016; 16: 17–29. 27018280 10.1111/ggi.12724

[60] Lehtonen JM, Forsstrom S, Bottani E, Viscomi C, Baris OR, Isoniemi H et al FGF21 is a biomarker for mitochondrial translation and mtDNA maintenance disorders. Neurology 2016; 87: 2290–9. 27794108 10.1212/WNL.0000000000003374 PMC5270510

[61] Steinbusch MM, Fang Y, Milner PI, Clegg PD, Young DA, Welting TJ et al Serum snoRNAs as biomarkers for joint ageing and post traumatic osteoarthritis. Sci Rep 2017; 7: 43558. 28252005 10.1038/srep43558 PMC5333149

[62] Loeser RF, Pathmasiri W, Sumner SJ, McRitchie S, Beavers D, Saxena P et al Association of urinary metabolites with radiographic progression of knee osteoarthritis in overweight and obese adults: an exploratory study. Osteoarthritis Cartilage 2016; 24: 1479–86. 27012755 10.1016/j.joca.2016.03.011 PMC4955662

[63] Ahmed U, Anwar A, Savage RS, Thornalley PJ, Rabbani N Protein oxidation, nitration and glycation biomarkers for early-stage diagnosis of osteoarthritis of the knee and typing and progression of arthritic disease. Arthritis Res Ther 2016; 18: 250. [journal article]. 27788684 10.1186/s13075-016-1154-3 PMC5081671

[64] Hobert O Gene regulation by transcription factors and microRNAs. Science 2008; 319: 1785–6. 18369135 10.1126/science.1151651

[65] McCarthy JJ MicroRNA-206: the skeletal muscle-specific myomiR. Biochim Biophys Acta 2008; 1779: 682–91. 18381085 10.1016/j.bbagrm.2008.03.001 PMC2656394

[66] Hackl M, Heilmeier U, Weilner S, Grillari J Circulating microRNAs as novel biomarkers for bone diseases - Complex signatures for multifactorial diseases? Mol Cell Endocrinol 2016; 432: 83–95. 26525415 10.1016/j.mce.2015.10.015

[67] Heilmeier U, Hackl M, Skalicky S, Weilner S, Schroeder F, Vierlinger K et al Serum microRNAs are indicative of skeletal fractures in postmenopausal women with and without type 2 diabetes and influence osteogenic and adipogenic differentiation of adipose-tissue derived mesenchymal stem cells in vitro. J Bone Miner Res 2016; 31: 2173–92. 27345526 10.1002/jbmr.2897

[68] Weilner S, Skalicky S, Salzer B, Keider V, Wagner M, Hildner F et al Differentially circulating miRNAs after recent osteoporotic fractures can influence osteogenic differentiation. Bone 2015; 79: 43–51. 26026730 10.1016/j.bone.2015.05.027

[69] Weilner S, Schraml E, Wieser M, Messner P, Schneider K, Wassermann K et al Secreted microvesicular miR-31 inhibits osteogenic differentiation of mesenchymal stem cells. Aging Cell 2016; 15: 744–54. 27146333 10.1111/acel.12484 PMC4933673

[70] Hackl M, Brunner S, Fortschegger K, Schreiner C, Micutkova L, Muck C et al miR-17, miR-19b, miR-20a, and miR-106a are down-regulated in human aging. Aging Cell 2010; 9: 291–6. 20089119 10.1111/j.1474-9726.2010.00549.x PMC2848978

[71] Li CJ, Cheng P, Liang MK, Chen YS, Lu Q, Wang JY et al MicroRNA-188 regulates age-related switch between osteoblast and adipocyte differentiation. J Cli Investig 2015; 125: 1509–22. 10.1172/JCI77716 PMC4396470 25751060

[72] Weilner S, Grillari-Voglauer R, Redl H, Grillari J, Nau T The role of microRNAs in cellular senescence and age-related conditions of cartilage and bone. Acta Orthop 2015; 86: 92–9. 25175665 10.3109/17453674.2014.957079 PMC4366666

[73] Balaskas P, Goljanek-Whysall K, Clegg P, Fang Y, Cremers A, Emans P et al MicroRNA profiling in cartilage ageing. Int J Genomics 2017; 2017: 2713725. 28890892 10.1155/2017/2713725 PMC5584353

[74] Cruz-Jentoft AJ, Baeyens JP, Bauer JM, Boirie Y, Cederholm T, Landi F et al Sarcopenia: European consensus on definition and diagnosis: report of the European Working Group on Sarcopenia in Older People. Age Ageing 2010; 39: 412–23. 20392703 10.1093/ageing/afq034 PMC2886201

[75] Janssen I, Heymsfield SB, Ross R Low relative skeletal muscle mass (sarcopenia) in older persons is associated with functional impairment and physical disability. J Am Geriatr Soc 2002; 50: 889–96. 12028177 10.1046/j.1532-5415.2002.50216.x

[76] Fosbol MO, Zerahn B Contemporary methods of body composition measurement. Clin Physiol Funct Imaging 2015; 35: 81–97. 24735332 10.1111/cpf.12152

[77] Goodman MJ, Ghate SR, Mavros P, Sen S, Marcus RL, Joy E et al Development of a practical screening tool to predict low muscle mass using NHANES 1999-2004. J Cachexia Sarcopenia Muscle 2013; 4: 187–97. 23673689 10.1007/s13539-013-0107-9 PMC3774922

[78] Prado CM, Siervo M, Mire E, Heymsfield SB, Stephan BC, Broyles S et al A population-based approach to define body-composition phenotypes. Am J Clin Nutr 2014; 99: 1369–77. 24760978 10.3945/ajcn.113.078576

[79] Janssen I, Heymsfield SB, Baumgartner RN, Ross R Estimation of skeletal muscle mass by bioelectrical impedance analysis. J Appl Physiol 2000; 89: 465–71. 10926627 10.1152/jappl.2000.89.2.465

[80] Yoshida D, Shimada H, Park H, Anan Y, Ito T, Harada A et al Development of an equation for estimating appendicular skeletal muscle mass in Japanese older adults using bioelectrical impedance analysis. Geriatr Gerontol Int 2014; 14: 851–7. 24450604 10.1111/ggi.12177

[81] Janssen I, Heymsfield SB, Baumgartner RN, Ross R Estimation of skeletal muscle mass by bioelectrical impedance analysis. J Appl Physiol 1985; 89: 465–71. 10.1152/jappl.2000.89.2.465 10926627

[82] Alizadehkhaiyat O, Hawkes DH, Kemp GJ, Howard A, Frostick SP Muscle strength and its relationship with skeletal muscle mass indices as determined by segmental bio-impedance analysis. Eur J Appl Physiol 2014; 114: 177–85. 24178819 10.1007/s00421-013-2764-y

[83] Yamada Y, Ikenaga M, Takeda N, Morimura K, Miyoshi N, Kiyonaga A et al Estimation of thigh muscle cross-sectional area by single- and multifrequency segmental bioelectrical impedance analysis in the elderly. J Appl Physiol 2014; 116: 176–82. 24114698 10.1152/japplphysiol.00772.2013

[84] Beaudart C, Reginster JY, Slomian J, Buckinx F, Dardenne N, Quabron A et al Estimation of sarcopenia prevalence using various assessment tools. Exp Gerontol 2015; 61: 31–7. 25449859 10.1016/j.exger.2014.11.014

[85] Morse CI, Thom JM, Davis MG, Fox KR, Birch KM, Narici MV Reduced plantarflexor specific torque in the elderly is associated with a lower activation capacity. Eur J Appl Physiol 2004; 92: 219–26. 15054662 10.1007/s00421-004-1056-y

[86] Puthucheary ZA, Rawal J, McPhail M, Connolly B, Ratnayake G, Chan P et al Acute skeletal muscle wasting in critical illness. J Am Med Assoc 2013; 310: 1591–600. 10.1001/jama.2013.278481 24108501

[87] Cooper C, Fielding R, Visser M, van Loon LJ, Rolland Y, Orwoll E et al Tools in the assessment of sarcopenia. Calcif Tissue Int 2013; 93: 201–10. 23842964 10.1007/s00223-013-9757-z PMC3744387

[88] Mitchell WK, Williams J, Atherton P, Larvin M, Lund J, Narici M Sarcopenia, dynapenia, and the impact of advancing age on human skeletal muscle size and strength; a quantitative review. Front Physiol 2012; 3: 260. 22934016 10.3389/fphys.2012.00260 PMC3429036

[89] Heymsfield SB, Adamek M, Gonzalez MC, Jia G, Thomas DM Assessing skeletal muscle mass: historical overview and state of the art. J Cachexia Sarcopenia Muscle 2014; 5: 9–18. 24532493 10.1007/s13539-014-0130-5 PMC3953319

[90] Ticinesi A, Meschi T, Narici MV, Lauretani F, Maggio M Muscle ultrasound and sarcopenia in older individuals: a clinical perspective. J Am Med Dir Assoc 2017; 18: 290–300. 28202349 10.1016/j.jamda.2016.11.013

[91] Narici MV, Binzoni T, Hiltbrand E, Fasel J, Terrier F, Cerretelli P *In vivo* human gastrocnemius architecture with changing joint angle at rest and during graded isometric contraction. J Physiol 1996; 496: 287–97. 8910216 10.1113/jphysiol.1996.sp021685 PMC1160844

[92] de Boer MD, Maganaris CN, Seynnes OR, Rennie MJ, Narici MV Time course of muscular, neural and tendinous adaptations to 23 day unilateral lower-limb suspension in young men. J Physiol 2007; 583: 1079–91. 17656438 10.1113/jphysiol.2007.135392 PMC2277190

[93] Seynnes OR, de Boer M, Narici MV Early skeletal muscle hypertrophy and architectural changes in response to high-intensity resistance training. J Appl Physiol 2007; 102: 368–73. 17053104 10.1152/japplphysiol.00789.2006

[94] Reeves ND, Maganaris CN, Narici MV Plasticity of dynamic muscle performance with strength training in elderly humans. Muscle Nerve 2005; 31: 355–64. 15654690 10.1002/mus.20275

[95] Narici MV, Maganaris CN, Reeves ND, Capodaglio P Effect of aging on human muscle architecture. J Appl Physiol 2003; 95: 2229–34. 12844499 10.1152/japplphysiol.00433.2003

[96] Ismail C, Zabal J, Hernandez HJ, Woletz P, Manning H, Teixeira C et al Diagnostic ultrasound estimates of muscle mass and muscle quality discriminate between women with and without sarcopenia. Front Physiol 2015; 6: 302. 26578974 10.3389/fphys.2015.00302 PMC4625057

[97] Narici MV, Campbell EL, McPhee J, Trisolino G, Seynnes OR, Conte M et al Muscle structural and functional changes with ageing, disuse and exercise (abstract presented at 41st European Muscle Conference, 1–5 Sept 2012, Rodes, Greece. J Muscle Res Cell Motil 2012; 33: 268–9.

[98] Noorkoiv M, Nosaka K, Blazevich AJ Assessment of quadriceps muscle cross-sectional area by ultrasound extended-field-of-view imaging. Eur J Appl Physiol 2010; 109: 631–9. 20191287 10.1007/s00421-010-1402-1

[99] Rosenberg JG, Ryan ED, Sobolewski EJ, Scharville MJ, Thompson BJ, King GE Reliability of panoramic ultrasound imaging to simultaneously examine muscle size and quality of the medial gastrocnemius. Muscle Nerve 2014; 49: 736–40. 24038069 10.1002/mus.24061

[100] Fukumoto Y, Ikezoe T, Yamada Y, Tsukagoshi R, Nakamura M, Mori N et al Skeletal muscle quality assessed from echo intensity is associated with muscle strength of middle-aged and elderly persons. Eur J Appl Physiol 2012; 112: 1519–25. 21847576 10.1007/s00421-011-2099-5

[101] Cadore EL, Izquierdo M, Conceicao M, Radaelli R, Pinto RS, Baroni BM et al Echo intensity is associated with skeletal muscle power and cardiovascular performance in elderly men. Exp Gerontol 2012; 47: 473–8. 22525196 10.1016/j.exger.2012.04.002

[102] Hansen M, Kongsgaard M, Holm L, Skovgaard D, Magnusson SP, Qvortrup K et al Effect of estrogen on tendon collagen synthesis, tendon structural characteristics, and biomechanical properties in postmenopausal women. J Appl Physiol 2009; 106: 1385–93. 18927264 10.1152/japplphysiol.90935.2008

[103] Hinsley H, Nicholls A, Daines M, Wallace G, Arden N, Carr A Classification of rotator cuff tendinopathy using high definition ultrasound. Muscles Ligaments Tendons J 2014; 4: 391–7. 25489559 PMC4241433

[104] Slane LC, Thelen DG Achilles tendon displacement patterns during passive stretch and eccentric loading are altered in middle-aged adults. Med Eng Phys 2015; 37: 712–6. 25962378 10.1016/j.medengphy.2015.04.004 PMC4478106

[105] Hansen P, Bojsen-Moller J, Aagaard P, Kjaer M, Magnusson SP Mechanical properties of the human patellar tendon, in vivo. Clin Biomech 2006; 21: 54–8. 10.1016/j.clinbiomech.2005.07.008 16183183

[106] Maganaris CN, Narici MV, Reeves ND In vivo human tendon mechanical properties: effect of resistance training in old age. J Musculoskelet Neuronal Interact 2004; 4: 204–8. 15615128

[107] Narici MV, Maffulli N, Maganaris CN Ageing of human muscles and tendons. Disabil Rehabil 2008; 30: 1548–54. 18608375 10.1080/09638280701831058

[108] Onambele GL, Narici MV, Maganaris CN Calf muscle-tendon properties and postural balance in old age. J Appl Physiol 2006; 100: 2048–56. 16455811 10.1152/japplphysiol.01442.2005

[109] Onambele GL, Narici MV, Rejc E, Maganaris CN Contribution of calf muscle-tendon properties to single-leg stance ability in the absence of visual feedback in relation to ageing. Gait Posture 2007; 26: 343–8. 17129729 10.1016/j.gaitpost.2006.09.081

[110] Reeves ND, Maganaris CN, Narici MV Effect of strength training on human patella tendon mechanical properties of older individuals. J Physiol 2003; 548: 971–81. 12626673 10.1113/jphysiol.2002.035576 PMC2342903

[111] Abd El Monaem SM, Hashaad NI, Ibrahim NH Correlations between ultrasonographic findings, clinical scores, and depression in patients with knee osteoarthritis. Eur J Rheumatol 2017; 4: 205–9. 28983413 10.5152/eurjrheum.2017.160097 PMC5621843

[112] Faisal A, Ng SC, Goh SL, Lai KW Knee cartilage segmentation and thickness computation from ultrasound images. Med Biol Eng Comput 2018; 56: 657–69. 28849317 10.1007/s11517-017-1710-2

[113] Joseph GB, McCulloch CE, Nevitt MC, Heilmeier U, Nardo L, Lynch JA et al A reference database of cartilage 3 T MRI T2 values in knees without diagnostic evidence of cartilage degeneration: data from the osteoarthritis initiative. Osteoarthritis Cartilage 2015; 23: 897–905. 25680652 10.1016/j.joca.2015.02.006 PMC4444394

[114] Wei W, Lambach B, Jia G, Kaeding C, Flanigan D, Knopp MV A Phase I clinical trial of the knee to assess the correlation of gagCEST MRI, delayed gadolinium-enhanced MRI of cartilage and T2 mapping. Eur J Radiol 2017; 90: 220–4. 28583638 10.1016/j.ejrad.2017.02.030

[115] Goto H, Iwama Y, Fujii M, Aoyama N, Kubo S, Kuroda R et al A preliminary study of the T1rho values of normal knee cartilage using 3T-MRI. Eur J Radiol 2012; 81: e796–803. 22525597 10.1016/j.ejrad.2012.03.022

[116] Shao H, Chang EY, Pauli C, Zanganeh S, Bae W, Chung CB et al UTE bi-component analysis of T2* relaxation in articular cartilage. Osteoarthritis Cartilage 2016; 24: 364–73. 26382110 10.1016/j.joca.2015.08.017 PMC4898889

[117] Guralnik JM, Simonsick EM, Ferrucci L, Glynn RJ, Berkman LF, Blazer DG et al A short physical performance battery assessing lower extremity function: association with self-reported disability and prediction of mortality and nursing home admission. J Gerontol Med Sci 1994; 49: M85–94. 10.1093/geronj/49.2.m85 8126356

[118] Freiberger E, de Vreede P, Schoene D, Rydwik E, Mueller V, Frandin K et al Performance-based physical function in older community-dwelling persons: a systematic review of instruments. Age Ageing 2012; 41: 712–21. 22885845 10.1093/ageing/afs099

[119] Ditroilo M, Forte R, Benelli P, Gambarara D, De Vito G Effects of age and limb dominance on upper and lower limb muscle function in healthy males and females aged 40–80 years. J Sports Sci 2010; 28: 667–77. 20397097 10.1080/02640411003642098

[120] Tieland M, Verdijk LB, de Groot LC, van Loon LJ Handgrip strength does not represent an appropriate measure to evaluate changes in muscle strength during an exercise intervention program in frail older people. Int J Sport Nutr Exerc Metab 2015; 25: 27–36. 24903908 10.1123/ijsnem.2013-0123

[121] Bento PC, Pereira G, Ugrinowitsch C, Rodacki AL Peak torque and rate of torque development in elderly with and without fall history. Clin Biomech 2010; 25: 450–4. 10.1016/j.clinbiomech.2010.02.002 20350773

[122] Macaluso A, De Vito G Comparison between young and older women in explosive power output and its determinants during a single leg-press action after optimisation of load. Eur J Appl Physiol 2003; 90: 458–63. 12883896 10.1007/s00421-003-0866-7

[123] Aagaard P, Simonsen EB, Andersen JL, Magnusson P, Dyhre-Poulsen P Increased rate of force development and neural drive of human skeletal muscle following resistance training. J Appl Physiol 2002; 93: 1318–26. 12235031 10.1152/japplphysiol.00283.2002

[124] Quinlan JI, Maganaris CN, Franchi MV, Smith K, Atherton PJ, Szewczyk NJ et al Muscle and tendon contributions to reduced rate of torque development in healthy older males. J Gerontol A Biol Sci Med Sci 2018; 73: 539–45. 28977366 10.1093/gerona/glx149 PMC5861887

[125] Lourenco RA, Perez-Zepeda M, Gutierrez-Robledo L, Garcia-Garcia FJ, Rodriguez Manas L Performance of the European Working Group on Sarcopenia in Older People algorithm in screening older adults for muscle mass assessment. Age Ageing 2015; 44: 334–8. 25539836 10.1093/ageing/afu192

[126] Aleman Mateo H, Lee SY, Javed F, Thornton J, Heymsfield SB, Pierson RN et al Elderly Mexicans have less muscle and greater total and truncal fat compared to African-Americans and Caucasians with the same BMI. J Nutr Health Aging 2009; 13: 919–23. 19924354 10.1007/s12603-009-0252-1 PMC2819676

[127] Doldo NA, Delmonico MJ, Bailey JA, Hand BD, Kostek MC, Rabon-Stith KM et al Muscle-power quality: does sex or race affect movement velocity in older adults? J Aging Phys Act 2006; 14: 411–22. 17215559 10.1123/japa.14.4.411

[128] Janssen I, Heymsfield SB, Wang ZM, Ross R Skeletal muscle mass and distribution in 468 men and women aged 18-88 yr. J Appl Physiol 2000; 89: 81–8. 10904038 10.1152/jappl.2000.89.1.81

[129] Teunis T, Lubberts B, Reilly BT, Ring D A systematic review and pooled analysis of the prevalence of rotator cuff disease with increasing age. J Shoulder Elbow Surg 2014; 23: 1913–21. 25441568 10.1016/j.jse.2014.08.001

[130] Robinson JM, Cook JL, Purdam C, Visentini PJ, Ross J, Maffulli N et al The VISA-A questionnaire: a valid and reliable index of the clinical severity of Achilles tendinopathy. Br J Sports Med 2001; 35: 335–41. 11579069 10.1136/bjsm.35.5.335 PMC1724384

[131] Breckenridge JD, McAuley JH Shoulder Pain and Disability Index (SPADI). J Physiother 2011; 57: 197. 21843839 10.1016/S1836-9553(11)70045-5

[132] van Schooten KS, Pijnappels M, Rispens SM, Elders PJ, Lips P, van Dieen JH Ambulatory fall-risk assessment: amount and quality of daily-life gait predict falls in older adults. J Gerontol A Biol Sci Med Sci 2015; 70: 608–15. 25568095 10.1093/gerona/glu225

[133] Yardley L, Beyer N, Hauer K, Kempen G, Piot-Ziegler C, Todd C Development and initial validation of the Falls Efficacy Scale-International (FES-I). Age Ageing 2005; 34: 614–9. 16267188 10.1093/ageing/afi196

[134] Powell LE, Myers AM The activities-specific balance confidence (ABC) scale. J Gerontol Biol Sci Med Sci 1995; 50A: M28–34. 10.1093/gerona/50a.1.m28 7814786

[135] Pasma JH, Bijlsma AY, van der Bij MD, Arendzen JH, Meskers CG, Maier AB Age-related differences in quality of standing balance using a composite score. Gerontol 2014; 60: 306–14. 10.1159/000357406 24968882

[136] Blaszczyk JW, Lowe DL, Hansen PD Ranges of postural stability and their changes in the elderly. Gait Posture 1994; 2: 11–7.

[137] Cofre Lizama LE, Pijnappels M, Rispens SM, Reeves NP, Verschueren SM, van Dieen JH Mediolateral balance and gait stability in older adults. Gait Posture 2015; 42: 79–84. 25953503 10.1016/j.gaitpost.2015.04.010

[138] Toebes MJ, Hoozemans MJ, Furrer R, Dekker J, van Dieen JH Local dynamic stability and variability of gait are associated with fall history in elderly subjects. Gait Posture 2012; 36: 527–31. 22748312 10.1016/j.gaitpost.2012.05.016

[139] Sawers A, Ting LH Beam walking can detect differences in walking balance proficiency across a range of sensorimotor abilities. Gait Posture 2015; 41: 619–23. 25648493 10.1016/j.gaitpost.2015.01.007

[140] Geerse DJ, Coolen BH, Roerdink M Kinematic validation of a multi-Kinect v2 instrumented 10-meter walkway for quantitative gait assessments. PLoS One 2015; 10: e0139913. 26461498 10.1371/journal.pone.0139913 PMC4603795

[141] Houdijk H, van Ooijen MW, Kraal JJ, Wiggerts HO, Polomski W, Janssen TW et al Assessing gait adaptability in people with a unilateral amputation on an instrumented treadmill with a projected visual context. Phys Ther 2012; 92: 1452–60. 22836005 10.2522/ptj.20110362

[142] Toebes MJ, Hoozemans MJM, Dekker J, van Dieen JH Associations between measures of gait stability, leg strength and fear of falling. Gait Posture 2015; 41: 76–80. 25242294 10.1016/j.gaitpost.2014.08.015

[143] Allet L, Kim H, Ashton-Miller J, De Mott T, Richardson JK Frontal plane hip and ankle sensorimotor function, not age, predicts unipedal stance time. Muscle Nerve 2012; 45: 578–85. 22431092 10.1002/mus.22325 PMC3313445

[144] Bijlsma AY, Pasma JH, Lambers D, Stijntjes M, Blauw GJ, Meskers CG et al Muscle strength rather than muscle mass is associated with standing balance in elderly outpatients. J Am Med Dir Assoc 2013; 14: 493–8. 23540951 10.1016/j.jamda.2013.02.001

[145] Granacher U, Gollhofer A, Hortobagyi T, Kressig RW, Muehlbauer T The importance of trunk muscle strength for balance, functional performance, and fall prevention in seniors: a systematic review. Sports Med 2013; 43: 627–41. 23568373 10.1007/s40279-013-0041-1

[146] Arvin M, van Dieën JH, Faber GS, Pijnappels M, Hoozemans MJM, Verschueren SM Hip abductor neuromuscular capacity: a limiting factor in mediolateral balance control in older adults? Clin Biomech 2016; 37: 27–33. 10.1016/j.clinbiomech.2016.05.015 27286555

[147] Wu G The relation between age-related changes in neuromusculoskeletal system and dynamic postural responses to balance disturbance. J Gerontol Biol Sci Med Sci 1998; 53: M320–6. 10.1093/gerona/53a.4.m320 18314573

[148] Rijken NH, van Engelen BG, de Rooy JW, Geurts AC, Weerdesteyn V Trunk muscle involvement is most critical for the loss of balance control in patients with facioscapulohumeral muscular dystrophy. Clin Biomech 2014; 29: 855–60. 10.1016/j.clinbiomech.2014.07.008 25156185

[149] Pijnappels M, van der Burg PJ, Reeves ND, van Dieen JH Identification of elderly fallers by muscle strength measures. Eur J Appl Physiol 2008; 102: 585–92. 18071745 10.1007/s00421-007-0613-6 PMC2226001

[150] Althoff T, Sosic R, Hicks JL, King AC, Delp SL, Leskovec J Large-scale physical activity data reveal worldwide activity inequality. Nature 2017; 547: 336–9. 28693034 10.1038/nature23018 PMC5774986

[151] Del Din S, Godfrey A, Mazza C, Lord S, Rochester L Free-living monitoring of Parkinson’s disease: lessons from the field. Mov Disord 2016; 31: 1293–313. 27452964 10.1002/mds.26718

[152] Del Din S, Godfrey A, Galna B, Lord S, Rochester L Free-living gait characteristics in ageing and Parkinson’s disease: impact of environment and ambulatory bout length. J Neuroeng Rehabil 2016; 13: 46. 27175731 10.1186/s12984-016-0154-5 PMC4866360

[153] Del Din S, Godfrey A, Rochester L Validation of an accelerometer to quantify a comprehensive battery of gait characteristics in healthy older adults and Parkinson’s disease: toward clinical and at home use. IIEEE J Biomed Health Inform 2016; 20: 838–47. 10.1109/JBHI.2015.2419317 25850097

[154] Lord S, Galna B, Rochester L Moving forward on gait measurement: toward a more refined approach. Mov Disord 2013; 28: 1534–43. 24132841 10.1002/mds.25545

[155] Sanders JL, Minster RL, Barmada MM, Matteini AM, Boudreau RM, Christensen K et al Heritability of and mortality prediction with a longevity phenotype: the healthy aging index. J Gerontol Biol Sci Med Sci 2014; 69: 479–85. 10.1093/gerona/glt117 PMC3968826 23913930

[156] Collerton J, Davies K, Jagger C, Kingston A, Bond J, Eccles MP et al Health and disease in 85 year olds: baseline findings from the Newcastle 85+ cohort study. Br Med J 2009; 339: b4904. 20028777 10.1136/bmj.b4904 PMC2797051

[157] Lopez-Otin C, Blasco MA, Partridge L, Serrano M, Kroemer G The hallmarks of aging. Cell 2013; 153: 1194–217. 23746838 10.1016/j.cell.2013.05.039 PMC3836174

[158] Horvath S DNA methylation age of human tissues and cell types. Genome Biol 2013; 14: R115. 24138928 10.1186/gb-2013-14-10-r115 PMC4015143

[159] Chen BH, Marioni RE, Colicino E, Peters MJ, Ward-Caviness CK, Tsai PC et al DNA methylation-based measures of biological age: meta-analysis predicting time to death. Aging. 2016; 8: 1844–65. 27690265 10.18632/aging.101020 PMC5076441

[160] Ribel-Madsen R, Fraga MF, Jacobsen S, Bork-Jensen J, Lara E, Calvanese V et al Genome-wide analysis of DNA methylation differences in muscle and fat from monozygotic twins discordant for type 2 diabetes. PLoS One 2012; 7: e51302. 23251491 10.1371/journal.pone.0051302 PMC3519577

[161] Vidal-Bralo L, Lopez-Golan Y, Mera-Varela A, Rego-Perez I, Horvath S, Zhang Y et al Specific premature epigenetic aging of cartilage in osteoarthritis. Aging. 2016; 8: 2222–31. 27689435 10.18632/aging.101053 PMC5076459

[162] Olivieri F, Capri M, Bonafe M, Morsiani C, Jung HJ, Spazzafumo L et al Circulating miRNAs and miRNA shuttles as biomarkers: Perspective trajectories of healthy and unhealthy aging. Mech Ageing Dev 2017; 165: 162–70. 27986629 10.1016/j.mad.2016.12.004 PMC5481482

[163] Jylhava J, Pedersen NL, Hagg S Biological age predictors. EBioMedicine 2017; 21: 29–36. 28396265 10.1016/j.ebiom.2017.03.046 PMC5514388

[164] Burkle A, Moreno-Villanueva M, Bernhard J, Blasco M, Zondag G, Hoeijmakers JH et al MARK-AGE biomarkers of ageing. Mech Ageing Dev 2015; 151: 2–12. 25818235 10.1016/j.mad.2015.03.006

[165] Murray CJ, Vos T, Lozano R, Naghavi M, Flaxman AD, Michaud C et al Disability-adjusted life years (DALYs) for 291 diseases and injuries in 21 regions, 1990–2010: a systematic analysis for the Global Burden of Disease Study 2010. Lancet 2012; 380: 2197–223. 23245608 10.1016/S0140-6736(12)61689-4

[166] Laurin D, Verreault R, Lindsay J, MacPherson K, Rockwood K Physical activity and risk of cognitive impairment and dementia in elderly persons. Arch Neurol 2001; 58: 498–504. 11255456 10.1001/archneur.58.3.498

[167] Lee IM, Shiroma EJ, Lobelo F, Puska P, Blair SN, Katzmarzyk PT et al Effect of physical inactivity on major non-communicable diseases worldwide: an analysis of burden of disease and life expectancy. Lancet 2012; 380: 219–29. 22818936 10.1016/S0140-6736(12)61031-9 PMC3645500

